# Enhancement of anti‐sarcoma immunity by NK cells engineered with mRNA for expression of a EphA2‐targeted CAR

**DOI:** 10.1002/ctm2.70140

**Published:** 2025-01-06

**Authors:** Pui Yeng Lam, Natacha Omer, Josh K. M. Wong, Cui Tu, Louisa Alim, Gustavo R. Rossi, Maria Victorova, Hannah Tompkins, Cheng‐Yu Lin, Ahmed M. Mehdi, Amos Choo, Melissa R. Elliott, Elaina Coleborn, Jane Sun, Timothy Mercer, Orazio Vittorio, Lachlan J. Dobson, Alexander D. McLellan, Andrew Brooks, Zewen Kelvin Tuong, Seth W. Cheetham, Wayne Nicholls, Fernando Souza‐Fonseca‐Guimaraes

**Affiliations:** ^1^ Frazer Institute, Faculty of Medicine The University of Queensland Woolloongabba Queensland Australia; ^2^ Queensland Children's Hospital Brisbane Queensland Australia; ^3^ Australian Institute for Bioengineering and Nanotechnology University of Queensland St Lucia Queensland Australia; ^4^ BASE Facility University of Queensland St Lucia Queensland Australia; ^5^ Queensland Cyber Infrastructure Foundation Ltd (QCIF) Facility for Advanced Bioinformatics St Lucia Queensland Australia; ^6^ Ian Frazer Centre for Children's Immunotherapy Research, Child Health Research Centre, Faculty of Medicine The University of Queensland Brisbane Queensland Australia; ^7^ School of Biomedical Sciences, Faculty of Medicine and Health University of New South Wales Sydney New South Wales Australia; ^8^ Department of Microbiology and Immunology The University of Otago Dunedin New Zealand

**Keywords:** CAR‐NK cell therapy, EphA2, Ewing sarcoma, immunotherapy, osteosarcoma, paediatric sarcomas, rhabdomyosarcoma

## Abstract

**Background:**

Paediatric sarcomas, including rhabdomyosarcoma, Ewing sarcoma and osteosarcoma, represent a group of malignancies that significantly contribute to cancer‐related morbidity and mortality in children and young adults. These cancers share common challenges, including high rates of metastasis, recurrence or treatment resistance, leading to a 5‐year survival rate of approximately 20% for patients with advanced disease stages. Despite the critical need, therapeutic advancements have been limited over the past three decades. The advent of chimeric antigen receptor (CAR)‐based immunotherapies offers a promising avenue for novel treatments. However, CAR‐T cells have faced significant challenges and limited success in treating solid tumours due to issues such as poor tumour infiltration, immunosuppressive tumour microenvironments and off‐target effects. In contrast, the adaptation of CAR technology for natural killer (NK) cells has demonstrated potential in both haematological and solid tumours, suggesting a new therapeutic strategy for paediatric sarcomas.

**Methods:**

This study developed and validated a novel CAR‐NK cell therapy targeting the ephrin type‐A receptor‐2 (EphA2) antigen, which is highly expressed in various paediatric sarcomas.

**Results:**

CAR expression was successfully detected on the surface of NK cells post‐electroporation, indicating successful transfection. Significantly, EphA2‐specific CAR‐NK cells demonstrated enhanced cytotoxic activity against several paediatric sarcoma cell lines in vitro, including those of rhabdomyosarcoma, Ewing sarcoma and osteosarcoma, compared to unmodified NK cells. Transient messenger RNA (mRNA) transfection of NK cells is a safe approach in genetic engineering, with further chemical modifications to mRNA enhancing stability of temporal EphA2‐CAR expression in NK cells, thereby promoting prolonged protein expression. Additionally, in vivo EphA2‐CAR‐NK cells showed promising anti‐cancer activity in rhabdomyosarcoma and osteosarcoma mouse models.

**Conclusions:**

The study provides a foundational basis for the clinical evaluation of EphA2‐targeted CAR‐NK cell therapy across a spectrum of paediatric sarcomas. The enhanced anti‐tumour effects observed in vitro/vivo suggests potential for improved therapeutic outcomes in hard‐to‐cure paediatric sarcomas.

**Key points:**

- Addressing unmet clinical needs in paediatric Sarcomas. Paediatric sarcomas, including rhabdomyosarcoma, Ewing sarcoma, and osteosarcoma, exhibit poor survival rates in advanced disease stages. The lack of significant therapeutic progress over the past three decades necessitates innovative treatment approaches.
- Advancing immunotherapy with CAR‐NK cells. Natural killer (NK) cells modified with chimeric antigen receptors (CARs) represent a promising strategy to overcome the limitations of CAR‐T cells, particularly in solid tumours. CAR‐NK cells are associated with enhanced tumour targeting, reduced off‐target effects, and improved safety profiles.
- EphA2 as a therapeutic target. EphA2, a receptor overexpressed in multiple paediatric sarcomas, is identified as a viable target for CAR‐based immunotherapy due to its critical role in tumour progression and angiogenesis.
- Innovations in mRNA‐based engineering. This study demonstrates the feasibility of transient mRNA transfection to engineer NK cells for CAR expression, offering a non‐integrative and safer alternative to viral transduction. Enhancements in mRNA stability through chemical modifications, can further optimise protein expression.
- Preclinical efficacy of EphA2‐CAR NK cells. EphA2‐specific CAR‐NK cells exhibit superior cytotoxicity against sarcoma cell lines in vitro and demonstrate significant anti‐tumour activity in in vivo mouse models of rhabdomyosarcoma and osteosarcoma.
- Clinical translation potential. The findings establish a strong preclinical rationale for the clinical evaluation of EphA2‐targeted CAR‐NK therapy as a novel immunotherapeutic option for paediatric sarcomas.
- Future research directions: Combining EphA2‐CAR NK cells with immune checkpoint inhibitors or other immunomodulatory agents could further enhance therapeutic outcomes and durability. Advanced preclinical models mimicking human tumour microenvironments are needed to refine and optimise this therapeutic approach.

## Graphical abstract

- Human NK cells can be efficiently transfected with mRNA to safely express an EphA2‐CAR without genomic alterations.
- EphA2‐targeted CAR‐NK cells exhibit enhanced cytotoxicity against paediatric sarcomas, including rhabdomyosarcoma, Ewing sarcoma and osteosarcoma.
- This study demonstrate the potential of CAR‐NK cell therapies to improve outcomes in hard‐to‐cure paediatric sarcomas and provides a foundation for their clinical evaluation.

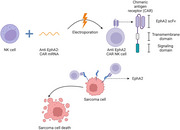

## 1 INTRODUCTION

Sarcomas are an aggressive group of cancers originating from mesenchymal tissues.^1^ Sarcomas can arise from bone (most commonly osteosarcoma [OS] and Ewing sarcoma [EWS]) and from soft tissue (most commonly rhabdomyosarcoma [RMS]).^2^ Patients with metastatic, relapsed or refractory paediatric sarcoma have a dismal prognosis with less than 20% maintaining long‐term survival.^3^ Furthermore, no significant improvement in patient outcome has been made over the last three decades with currently available therapies (surgery, radiation and chemotherapy).^4^, ^5^, ^6^ Hence, there is an urgent need for innovative therapeutic interventions, such as immunotherapy.

There is now strong evidence supporting the essential role of the innate immune system in controlling cancer development and progression, including natural killer (NK) cells.^7^ NK cells are a subset of lymphoid cells capable of recognising and eliminating infected, stressed and malignant cells.^8^ Their innate ability to detect and destroy cancer cells makes NK cells a crucial component of cancer surveillance and immune defense. Importantly, sarcomas as a group have been shown to be sensitive to the anti‐tumour effect of NK cells,^9^, ^10^ with high infiltration of NK cells in several subtypes of sarcoma tumours shown to correlate with improved patient survival,^11^, ^12^ indicating a suppressive role of NK cells in tumour initiation and progression.^12^ Recently, several studies have investigated the use of NK cells to exploit their anti‐tumour potential in preclinical models of sarcoma.^13^, ^14^ However, their use in the clinical field for sarcoma immunotherapy is still limited, with only one recent clinical trial reporting the results of testing autologous NK cell infusions against advanced sarcomas (NCT03941262).^15^, ^16^ A key challenge in NK cell therapy for solid tumours, including sarcomas, is the limited ability of NK cells to penetrate the tumour microenvironment effectively.^17^ To overcome this, improving NK cell delivery and persistence within tumours is critical, and requires new strategies such as using chimeric antigen receptors (CARs) to enhance NK cell trafficking and function.

Genetic engineering of CARs was first pioneered in T cells (CAR‐T cells), and this equipped T cells with enhanced ability to target and lyse tumour cells in an antigen‐specific manner. CAR‐T cells have been highly successful with impressive clinical efficacy in haematological malignancies.^18^ However, CAR‐T cell therapy has several significant limitations, primarily owing to the requirement for these cell products to be autologous. Consequently, the cost and time of manufacturing, and the possibility of severe side effects such as cytokine release syndrome (CRS) and neurotoxicity have restricted their widespread clinical application.^19^ In addition, to date CAR‐T cell approaches have failed to deliver a therapeutic product for hard‐to‐treat solid cancers largely due to the impact of the tumour microenvironment.^20^ In contrast, CAR‐NK therapy has seen success in early stage clinical trials for haematological cancers and early potential against solid tumours,^20^, ^21^, ^22^, ^23^ leading to several active and completed clinical trials involving CAR‐NK cells and are attracting an unprecedent industry appetite to this potential off‐the‐shelf cellular immunotherapy opportunity.^24^ Notably, CAR‐NK cells offer significant advantages over CAR‐T cells, including reduced alloreactivity, which enhances their potential as safer ‘off‐the‐shelf’ allogeneic therapies, a lower risk of graft‐versus‐host disease and toxic side effects including CRS, and an innate capacity to target tumour cells through NK cell receptor‐dependent mechanisms independently of CAR signalling.^25^ The first large‐scale phase I/II clinical trials using CAR‐NK cells was published in 2020 followed by a subsequent study in 2024. These studies provided convincing evidence of the exceptional safety and efficacy of CAR‐NK cells as a cancer immunotherapy for B‐cell lymphoma.^21^, ^26^ However, while the anti‐tumour effect of CAR‐NK cells has been demonstrated in in vitro and in vivo models of sarcoma,^27^, ^28^ the potential of engineered CAR‐NK cell therapy has not yet been translated to the clinic.

Genetically editing NK cells to express CARs involves significant manufacturing challenges, notably the difficulty of effectively transducing NK cells with viral vectors.^29^ Due to their unique biology, NK cells are resistant to genetic modification, making viral transduction less efficient and time consuming.^30^ Transduced CAR expression levels in primary NK cells have been reported to strongly vary between 20% and 70% but did not appear to affect specificity or cytotoxic functions against target‐positive tumour cells.^31^ Given that genetic modifications in CAR‐T cells have been associated with the potential risk of generating secondary cancers due to insertional mutagenesis,^32^ messenger RNA (mRNA) transfection has the additional benefit of long‐term safety by avoiding permanent genetic alterations. Therefore, the search for novel nanobiomaterials to engineer NK cells has sparked the interest in the cell therapy industry,^33^ and transiently engineering NK cells with mRNA presents a safer and more efficient alternative that allows careful optimisation for effective CAR expression without compromising cell viability and functionality, and patient safety. In concert, transiently engineering NK cells with mRNA presents a safer and more efficient alternative that allows careful optimisation for effective CAR expression without compromising cell viability and functionality. NK cells are highly amenable to electroporation for the delivery of payloads such as mRNA.^22^ The transient nature of mRNA allows for dose escalation during treatment and facilitates the natural removal of CARs through RNA degradation, thus eliminating the need for suicide or switch‐off mechanisms.^34^ Nevertheless, this approach provides a flexible and controlled method for CAR expression, which minimises long‐term risks and enables more precise management of therapy, which could be advantageous in rapidly adjusting treatment to patient‐specific needs or emerging clinical responses.

CARs have traditionally been designed to specifically target antigens that are overexpressed on cancer cells but rare on healthy tissue, thereby minimising CAR‐induced damage to normal cells.^35^, ^36^ In sarcomas, various antigens are present, with ephrin type‐A receptor‐2 (EphA2) being one of particular interest. EphA2 is a tyrosine kinase receptor for ephrin and has key functions in the developing embryo, but is mainly confined to proliferating epithelial cells in adults.^37^, ^38^ In paediatric sarcomas, EphA2 acts as a critical oncoprotein that regulates tumour angiogenesis and aggressiveness,^39^, ^40^ with EphA2 shown to be the most abundant upregulated cell surface receptor on OS cells.^41^ EphA2‐targeted CAR‐modified T cells have seen success in preclinical models of medulloblastoma,^42^ esophageal squamous cell carcinoma,^43^ EWS and OS,^44^ as well as preclinical models for glioblastoma.^45^, ^46^ Most recently, EphA2‐targeted CAR‐modified primary NK cells have demonstrated efficacy against preclinical models of EphA2 antigen‐positive head and neck squamous cell carcinoma (HNSCC),^47^ which highlights the applicability of EphA2‐targeting CAR‐modified NK cells for sarcoma immunotherapy. In this study, we correlated EphA2 expression and NK cell score and observed a survival benefit in sarcoma patients with highest NK cell infiltration, assessed various EphA2‐targeting CAR designs for enhancing NK cell functionality against paediatric sarcoma in vitro and in vivo, and investigated how chemical modifications of mRNA can improve the stability and longevity of CAR expression. These findings aim to provide insights into optimising CAR‐NK cell therapy for paediatric sarcomas by targeting EphA2, enhancing CAR stability, and provide insights into improving therapeutic efficacy of CAR‐NK cells for the outcomes of sarcoma patients.

## 2 MATERIAL AND METHODS

### 2.1 Cells

Sarcoma cell lines A673 (CRL‐1598), RD (CCL‐136), RH30 (CRL‐2061) and K‐562 lymphoblast cells (CCL‐243) were purchased from the American Type Culture Collection (ATCC), while MG63 (ATCC CRL‐1427), SaOS (ATCC HTB‐85) and U‐2 OS (U2OS, HTB‐96) were provided by A/Prof. Orazio Vittorio (Children's Cancer Institute & UNSW). Sarcoma cell lines are maintained in the following reagents purchased from Gibco: Dulbecco's modified eagle medium (DMEM) with 1× GlutaMAX supplement, 10% heat inactivated foetal bovine serum (HI‐FBS), 1% penicillin–streptomycin (PenStrep) and 1% non‐essential amino acids (NEAA). NK cell lines NK92 and Khyg1 cells were provided by Prof. Nicholas Huntington (Monash University) and maintained in Roswell Park Memorial Institute medium (RPMI) media (Gibco) with 1× GlutaMAX supplement, 10% HI‐FBS, 1% PenStrep, 10 mM HEPES and 300 IU/mL hIL‐2, NK MACS media (Miltenyi Biotec, Bergisch Gladbach) containing 10% human AB serum (Sigma‒Aldrich), 300 IU/mL hIL‐2 (Peprotech) and 5 ng/mL hIL‐15 (Peprotech) and RMPI media with 1× GlutaMAX, 10% HI‐FBS, 1% PenStrep and 1× NEAA, respectively. HEK293F cells were purchased from Thermo Fisher Scientific and cultured in Freestyle 293F Expression Media (Gibco) as per manufacturer's protocol. For in vivo tracking, stable green fluorescent protein (GFP)‐luciferase expressing MG63 and RD cell lines (RD‐/MG63‐GFP‐Luc^+^) were generated by transducing the cells with a lentiviral vector encoding firefly luciferase and EGFP—pLentipuro3/TO/V5‐GW/EGFP‐Firefly Luciferase (plasmid #119816, Addgene). GFP‐positive transduced cells were enriched by fluorescence‐activated cell sorting using a FACSAria II instrument (BD Biosciences).

Human primary NK cells were isolated from healthy donor buffy coats obtained from the Australian Red Cross Australia (agreement no. 23‐10QLD‐10). PBMCs were isolated using Ficoll Paque (Cytiva) and Leucosep (Greiner Bio‐One) centrifugation. Primary NK cells were purified from PBMCs using the MojoSort Human NK Cell Isolation Kit (BioLegend) according to manufacturer's protocol. To culture and expand human NK cells, 1 million/mL cells were cultured in complete NK cell culture media consisting of supplemented NK MACS media (Miltenyi Biotec), 5% heat inactivated human AB serum (Sigma‒Aldrich), 500 IU/mL hIL‐2 and 5 ng/mL hIL‐15 in G‐Rex 24 multi‐well cell culture plates (Wilson Wolf) in the presence of NK cell activation beads (Miltenyi Biotec) or in presence of CD86/4‐1BBL/mbIL‐15/mbIL‐21‐transfected K562 feeder cells^48^ at a 1:1 ratio (NK:feeder) and plates were incubated at 37°C with 5% CO_2_. Similarly, mononuclear cells from cord blood (CB) were plated in G‐Rex 24‐well plates (#80192 M—Wilson Wolf) at 2 million cells per well in 6 mL of complete NK MACS medium in presence of K562 feeder cells. Cells were maintained in complete NK cell culture media, with a half media change every 3–4 days and full media change every 7 days until utilised in experiments.

Feeder cells were obtained by transfecting K562 cells (ATCC) at the Genome Engineering Facility (Children's Medical Research Institute) with Sleeping Beauty plasmids encoding CD86, 4‐1BBL, mbIL‐21 and mbIL‐15. Plasmids were kindly gifted by Professor Alexander McLellan (University of Otago).^48^ The engineered K562 feeder cells were expanded in RPMI media, 10% HI‐FBS and GlutaMAX, irradiated (100 Gy) and cryopreserved in 10% dimethyl sulfoxide (DMSO) and 90% FBS for future utilisation.

### 2.2 Plasmid construction, in vitro transcription and stability testing

EphA2‐CAR constructs were designed to include a T7 promoter at the 5′ end, followed by a single‐chain variable fragment (scFv) specific to human EphA2 (clones 4H5,^49^ D2^50^ or D2‐1A7^51^), a flexible and commonly used CD8α hinge,^52^ a CD28‐CD3ζ signalling domain,^53^ with the inclusion of GFP sequence separated from the CAR construct by a 2A self‐cleaving peptide at the 3′ end. EphA2‐CAR‐GFP constructs were cloned into the plasmid pcDNA3.1 backbone and assembled using NEBuilder HiFi DNA Assembly kit (New England Biolabs [NEB]) according to manufacturer's instructions. Colony clones selected and purified using Wizard Plus SV Minipreps DNA Purification Systems kit (Promega) and all cloning steps were validated by restriction enzyme digestion and sequencing. HiScribe T7 ARCA mRNA Kit (NEB) and Monarch RNA Clean Up Kit (NEB) were used to in vitro transcribe mRNA from linearised DNA template and purify mRNA, respectively. Synthetic codon‐optimised and uridine‐modified EphA2‐CAR (4H5 clone) without GFP sequence and eGFP mRNA sequences were additionally produced by the University of Queensland BASE mRNA Facility. mRNA produced at the facility uses a pBASE vector for in vitro transcription (IVT) of mRNA that features the codon optimised coding sequence flanked by the human alpha globin 5′ UTR and AES‐mtRNR1 3′ UTR sequences, a 120 nt segmented polyA tail with adjacent BsaI for linearisation. IVT mRNA is purified and routinely verified using RNA ScreenTape on the Agilent 2200 TapeStation system (Agilent Technologies). BASE mRNA uses standard rNTPs (ATP, CTP, GTP), with UTP substituted with equimolar concentrations of the modified counterpart for N1‐methylpseudouridine (m1Ψ) or further modified with 10%, 25% or 50% adenosine‐5′‐(α‐thio)‐triphosphate (ATPαS; Jena Bioscience). Capping is performed co‐transcriptionally with the CleanCapAG reagent. For stability testing, mRNA samples with various nucleotide modifications were stored at 4°C or room temperature (RT) for 1 week. Concentration was measured using an Implen NanoPhotometer (Implen). mRNA integrity was measured using an Agilent TapeStation 4200 with the RNA ScreenTape assay (Agilent). Briefly, mRNA samples were diluted to 200 ng/µL, then 1 µL of sample was added to 5 µL of RNA ScreenTape Sample Buffer. Samples were then vortexed for 1 min using the IKA MS3 vortex (IKA) and denatured at 72°C for 3 min before running the RNA ScreenTape assay.

### 2.3 mRNA‐lipid nanoparticle production

The design, manufacture and formulation of lipid nanoparticle (LNP)‐containing mRNA was performed as per routine protocols based in Moderna Therapeutics formulations,^54^ used by the UQ BASE mRNA Facility.^55^ Cholesterol was obtained from Avanti Lipids. SM‐102, 1,2‐distearoyl‐*sn*‐glycero‐3‐PC (DSPC) and DMG‐PEG 2000 were obtained from Cayman Chemical. mRNA‐LNPs were prepared using the NanoAssemblr Ignite (Precision NanoSystems). mRNA was diluted in .1 M sodium acetate buffer at pH 4.0 (Fisher Bioreagents) to a final concentration of approximately 130 µg/mL. Lipids were prepared to at a total lipid concentration of 10 mg/mL in 200 proof ethanol (Sigma‒Aldrich) at a molar ratio of 50:38.5:10:1.5, SM‐102, cholesterol, DSPC and DMG‐PEG 2000, respectively. LNPs were formulated at a flow rate ratio of 3:1, aqueous to organic phase, and a total flow rate of 12 mL/min. Immediately post‐formulation, LNPs were dialysed overnight at 4°C in 1× tris‐buffered saline (TBS) using a 10 K MWCO Slide‐a‐Lyzer dialysis cassette (Thermo Fisher Scientific). Following dialysis, LNPs were concentrated to 1 mL using a 10 K MWCO Amicon Ultra‐15 centrifugal filter (Sigma‒Aldrich). LNPs were filtered using a .22 µM filter (FilterBio) and 10% sucrose was added before storage at ‒20°C. Size, polydispersity and zeta potential analysis was completed using a Zetasizer Ultra (Malvern Panalytical) on an LNP sample diluted 1:20 in UltraPure water (Thermo Fisher Scientific). Encapsulation efficiency and encapsulated concentration was determined using the Quant‐it RiboGreen RNA assay kit (Invitrogen Life Technologies).

### 2.4 mRNA transfections

For electroporation experiments, NK cell media was refreshed 24 h before transfection. NK cells from culture were resuspended in MaxCyte electroporation buffer (MaxCyte) containing RNAse inhibitor (Promega), and CAR mRNA (15 pmol/million cells or 200 µg/mL). For in vivo studies, controls with no mRNA added to the cells during transfections were used as the mock group. The MaxCyte STx system (MaxCyte) was used to electroporate cells using either NK‐2 or NK‐3 programs. Following electroporation, the cells were recovered for 20 min in a 37°C cell culture incubator in warm complete media. NK cells were then harvested at 24–72 h post‐electroporation for setting up cytotoxicity assays, flow cytometry analysis and for i.v. injections. For LNP‐based expression, NK cells were cultured in media containing LNP carrying mRNA at the same concentration used for electroporation experiments (15 pmol/million cells or 200 µg/mL).

### 2.5 Flow cytometry and microscopy analyses

For flow analysis, EphA2‐CAR‐GFP or GFP expression was determined 24–48 h post‐electroporation by staining 1 × 10^5^ cells with 1:1000 dilution of Zombie Aqua (BioLegend) for 10 min in phosphate‐buffered saline (PBS), followed by washing with 1× flow buffer (1× PBS containing 2% FBS and 2 mM ethylenediaminetetraacetic acid (EDTA)) once before staining with or without 1:50 dilution of Alexa Fluor 647‐conjugated anti‐G4S linker (E7O2V) rabbit monoclonal antibody (Cell Signaling Technology, Inc.) for 45 min at RT. For detection using Protein L, cells were stained either with .2 ng/µL of biotinylated Protein L (Genscript) for 45 min followed by a 1:400 dilution of V450 Streptavidin (BioLegend) for 45 min or 1:50 dilution of Protein L‐PE (Acrobiosystems) for 30 min at RT. For detection using goat anti‐human IgG‐PE (Jackson ImmunoResearch Laboratory), cells were stained with a 1:20 dilution of goat anti‐human IgG‐PE for 30 min at RT and washed twice with 1× flow buffer before flow analysis. For cell surface expression of EphA2 on paediatric sarcoma cell lines, the cells were washed in 1× flow buffer at 4°C before blocking with human Fc Block (1:100) for 15 min. Cells were then incubated with anti‐EphA2 antibody (phycoerythrin [PE] conjugate) clone SHM16 (BioLegend) for 1 h at RT and washed twice before flow analysis. For detection of functional activation in NK cells, electroporated NK cells were rested for 1 h at 37°C in complete NK MACS media containing 5 ng/mL of rhIL‐15. NK cells were then incubated for another 4 h in a cocktail containing 1:1000 dilution of Monensin, 1:1000 dilution of Brefeldin A with or without 1:500 dilution of Cell Activation Cocktail (BioLegend) and 1:100 dilution of anti‐human CD107a (BioLegend) in NK MACS media containing rhIL‐15 (5 ng/mL). Cells were stained for viability using Live/Dead Fixable Near‐IR (Invitrogen Life Technologies) and intracellular interferon‐gamma (IFN‐γ; BioLegend) and granzyme B (Becton Dickinson) production. All flow experiments were acquired on a BD LSRFortessa X‐20 (Becton Dickinson) or a CytoFLEX V2‐B5‐R3 (Beckman Coulter) flow cytometers and analysed with either FlowJo v10.8.1 (TreeStar) or FCS Express 7 Research Edition (De Novo Software) programs in a classical supervised manner (exclusion of debris/dead cells, gating of singlet, analysis of expression for markers) unless otherwise stated. For microscopy analysis of GFP expression after electroporation of EphA2‐CAR‐GFP or eGFP mRNA, cells were visualised using the Olympus IX73 fluorescent microscope and images were taken using the Olympus cellSens imaging software (Olympus). Images were processed using ImageJ (National Institutes of Health) system software. For analysis of GFP expression in lipofected HEK293F cells, flow cytometry was carried out on the Cytoflex V2‐B5‐R3 (Beckman Coulter) flow cytometer with 2–10 × 10^4^ single, viable cells/sample.

### 2.6 In vitro cytotoxicity assay

Calcein acetoxymethyl (AM) release‐based killing assay was adapted as previously described.^56^, ^57^, ^58^ In vitro Calcein AM cytotoxicity assays were performed to evaluate the cytotoxic capacity of the CAR‐modified NK cells over 4 h. Target cells were resuspended in NK cell cytotoxicity medium (phenol red‐free RPMI medium [Gibco] with 1 mM sodium pyruvate [Gibco], 1× NEAA [Gibco], 1× GlutaMAX [Gibco], 55 µM 2‐mercaptoethanol [Gibco] and 1% HEPES [Gibco]), at a final concentration of 10^6^ cells/mL and incubated with 15 µM Calcein‐AM (Invitrogen Life Technologies) for 30 min at 37°C with occasional shaking. Stained target cells were then washed twice with 10 mL PBS. NK cells (effector cells) were then serial diluted in NK cell cytotoxicity media (+10% FBS for NK92 cells or 5% AB serum for peripheral blood (PB)‐ or cord‐blood‐derived NK cells) in a 96‐well U‐bottom plate in various effector‐to‐target (E:T) ratios in triplicates. Target cells were then added to the plate at a concentration of 3 × 10^3^ cells per well and the plate was incubated at 37°C for 4 h. To determine the maximum and spontaneous Calcein release values, target cells alone were incubated with either NK cell cytotoxicity media containing 5% Triton X‐100 or NK cell cytotoxicity media alone, respectively. After 4 h, 100 µL supernatant was transferred to a 96‐well white flat bottom plate (Nunc, Thomas Scientific) and fluorescence was read using a CLARIOstar plate reader (BMG Labtech) (excitation 483‐10, emission 530‐10). Specific target lysis was calculated using the following formula: [(NK cell‐induced Calcein AM release − spontaneous Calcein AM release)/(maximum Calcein AM release − spontaneous Calcein AM release) × 100].

### 2.7 Bioinformatics analysis

RNA‐seq expression data and clinical characteristics for the primary tumour samples collected at the diagnosis stage of paediatric sarcoma patients were collected from the Therapeutically Applicable Research to Generate Effective Treatments (TARGET) database (Target‐OS database; https://www.cancer.gov/ccg/research/genome‐sequencing/target/usingtarget‐data; accession study: phs000468.v18.p7) and ArrayExpress database (https://www.ebi.ac.uk/biostudies/arrayexpress; accession ID E‐TABM‐1202 RMS dataset).^59^ The optimal cut‐off for the single factor in the survival analysis was determined using the data‐driven approach using the Conditional Inference Tree (CTREE) algorithm from the ‘party’ package (version 1.3.17) in R to create decision tree that optimises the separation between high and low groups for survival analysis.^60^ The survival analysis was performed using the ‘survival’ package (version 3.7.0), and Kaplan‒Meier (KM) curves were plotted using the ‘survminer’ package (version 0.4.9). Gene set scoring was performed to calculate glutathione metabolism score using the singscore approach.^61^ In short, genes are sorted in ascending order based on their transcript abundance. NK cell infiltration scoring method was obtained from ref.^62^ TARGET OS datasets are available without restrictions on their use in publications since 2019. We further processed single‐cell (sc)RNA‐seq data of infiltrating immune cells in OS from,^63^, ^64^ and healthy human femoral head^65^ as control, accessible from GEO database under the accession number GSE147390. Apart from the originally published quality control of these datasets, no additional quality controls were applied. The scRNA‐seq data were integrated using bbknn.^66^ Selected genes associated with NK cells exhaustion were visualised as violin plots using scanpy's^67^ sc.pl.violin function.

### 2.8 Mice

To support human NK cell survival and function, a human IL‐7 (hIL‐7) and IL‐15 (hL‐15) double knock‐in (KI) immunodeficient NOD SCID gamma (NSG) mice^68^, ^69^ backcrossed with an HLA‐A2 NSG mice^70^ were used (NSG/A2/hIL‐7/hIL‐15‐KI). Mice were bred and maintained onsite at the Translational Research Institute Biological Research Facility and housed under specific pathogen‐free conditions. Mice aged 6–8 weeks were used for stable engraftment of the human OS or RMS cell lines. All experiments were conducted following the animal ethics guidelines provided by the National Health and Medical Research Council of Australia and approved by the University of Queensland—Health Sciences Animal Ethics Committee (ethics number 2021/AE000294).

### 2.9 Xenogeneic sarcoma mouse models

To assess therapeutic efficacy of EphA2‐CAR‐NK cells against sarcoma in vivo, RMS (RD) and OS (MG63) cell lines were engrafted into the NSG/A2/hIL‐7/hIL‐15‐KI mice. For orthotopic RMS tumour inoculation, male and female mice were restrained with the hind limb in flexion and 2 × 10^6^ RD GFP^+^ Luc^+^ cell suspension were inoculated intra‐muscularly using a 30G insulin syringe needle, as described previously,^71^ after buprenorphine subcutaneous (s.c.) injection for analgesia (10 µg per 10 g of body weight). Mice were either left untreated (PBS only) or intravenously (i.v.) treated with 1 × 10^6^ mock or EphA2‐CAR transfected NK92 cells or primary NK cells when indicated. The mice lungs were inspected for metastasis ex vivo using the in vivo imaging system (IVIS) optical imager as previously described.^72^ For the s.c. OS model, 1 × 10^6^ MG63 GFP^+^ Luc^+^ cells were injected s.c. in the right flank of male mice. After 250 cGy of whole body γ‐irradiation on day ‒1, mice were treated with 3–5 × 10^6^ cells EphA2‐CAR mRNA or no mRNA (mock group) NK cells resuspended in PBS, or with PBS only (untreated) as indicated. Tumour engraftment and progression were evaluated by external caliper measurements of s.c tumours greatest longitudinal (length) and transverse (width) diameters 2–3 times a week, and on weekly IVIS imaging beginning 10 min after intraperitoneal injection of an aqueous solution of d‐luciferin potassium salt (150 mg/kg). Photons emitted from luciferase expressing cells were quantified using the Living Image 3.0 software program. Tumour volume was calculated by the ellipsoidal formula: *V* = 1/2 (length × width^2^). Mice were regularly monitored and sacrificed once tumours reached a size of 1 cm^3^ or if reaching a preestablished animal ethics wellbeing score of 3 in any category or a cumulative score of 15, and an overall survival was calculated as mice were sacrificed according to this criterion.

### 2.10 Statistical analyses

All other results are presented as mean ± SE. KM plots were used to analyse OS and RMS datasets from the TARGET program and E‐TABM‐1202 RMS dataset. For all other data, unpaired *t*‐test or Kruskal‒Wallis test was used to assess differences between two groups, one‐way or two‐way ANOVA and Tukey's multiple comparison test between two or more groups, and Gerhan‒Breslow‒Wilcoxon test for survival analysis (GraphPad Prism 10 Software). Statistical analysis in scRNAseq datasets (Wilcoxon rank sum test) of marker gene expression difference between NK cells was performed using scanpy's sc.tl.rank_genes_group function, where Benjamini‒Hochberg^73^ adjusted *p* < .05 was considered statistically significant.

## 3 RESULTS

### 3.1 Association of NK cell score and EphA2 expression with 5‐year survival in sarcoma patients using the TARGET database and RMS E‐TABM‐1202

**FIGURE 1:**
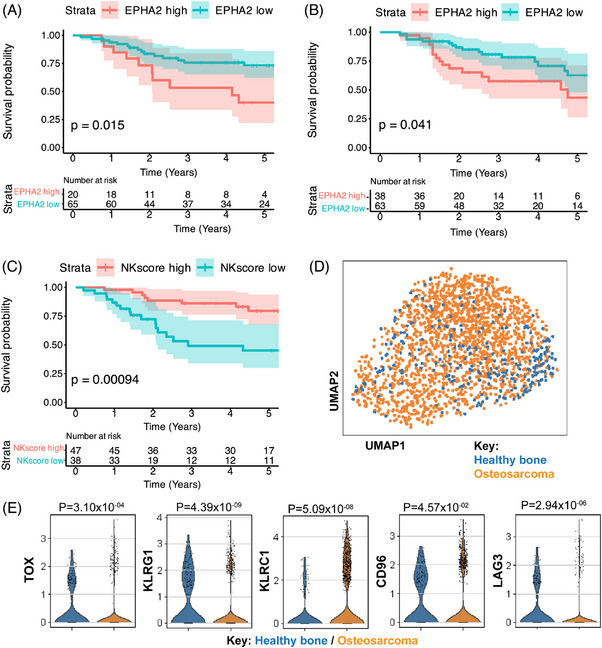
Ephrin type‐A receptor‐2 (EphA2) and natural killer (NK) cell infiltration correspond with sarcoma survival. The Kaplan‒Meier 5‐year survival curve (log‐rank test) from the TARGET‐OS (https://www.cancer.gov/ccg/research/genome‐sequencing/target/usingtarget‐data; accession study: phs000468.v18.p7) (A) and ArrayExpress database (https://www.ebi.ac.uk/biostudies/arrayexpress; accession ID E‐TABM‐1202 RMS) (B) datasets illustrates the prognosis for osteosarcoma or rhabdomyosarcoma (RMS) patients with high or low EphA2 gene expression. The Kaplan‒Meier 5‐year survival curves from the TARGET‐OS and dataset illustrates the prognosis for osteosarcoma patients with high and low NK cell infiltration (C). Uniform manifold approximation and projection (UMAP) analysis cluster show integration of osteosarcoma (OS) infiltration and healthy bone‐infiltrating NK cells from scRNAseq datasets, Refs.^63^, ^64^ and GSE147390, respectively (D), and each violin plot shows the normalised expression value of each gene on the *y*‐axis, split by the tissue on the *x*‐axis. Adjusted *p*‐values from Wilcoxon rank sum test are shown.

To validate the prognostic impact of NK cell infiltration and EphA2 expression in sarcoma patients, we utilised data from the TARGET‐OS program and transcriptional data obtained from the E‐TABM‐1202 dataset^59^ for RMS. These analyses included 84 OS patients with available gene expression profiles and clinical outcome data as well as 101 RMS patient samples with transcriptional gene expression profiles stratified by PAX3/FOXO1 Fusion Gene Status. Our analyses indicate that higher EphA2 expression are associated with significantly worse 5‐year survival rates in OS (Figure 1A) and RMS patients (Figure 1B) as compared to patients with lower EphA2 expression, suggesting that this receptor is a promising target for therapeutic intervention in these cancers. We further explored NK cell infiltration using a curated scoring method,^62^ and observed that OS patients with higher NK cell scores also have the best survival rates (Figure 1C). This led us to further analyse intratumoural NK cells using scRNAseq datasets, where we compared NK cells infiltrating OS and healthy femoral head as control (Figure 1D) and verified that OS‐infiltrating NK cells had elevated expression of the transcriptional factor TOX, and inhibitory markers such as CD96, KLRG1, KLRC1 and LAG3 as compared to control bone‐infiltrating cells (Figure 1E), indicating an exhausted phenotype. This exhaustion could potentially be reversed by equipping tumour‐infiltrating NK cells with enhancement‐of‐function genes such as a EphA2‐CAR and underscore the potential for developing a novel immunotherapeutic strategy for paediatric sarcomas.

### 3.2 Validation of CAR expression in NK cells

**FIGURE 2:**
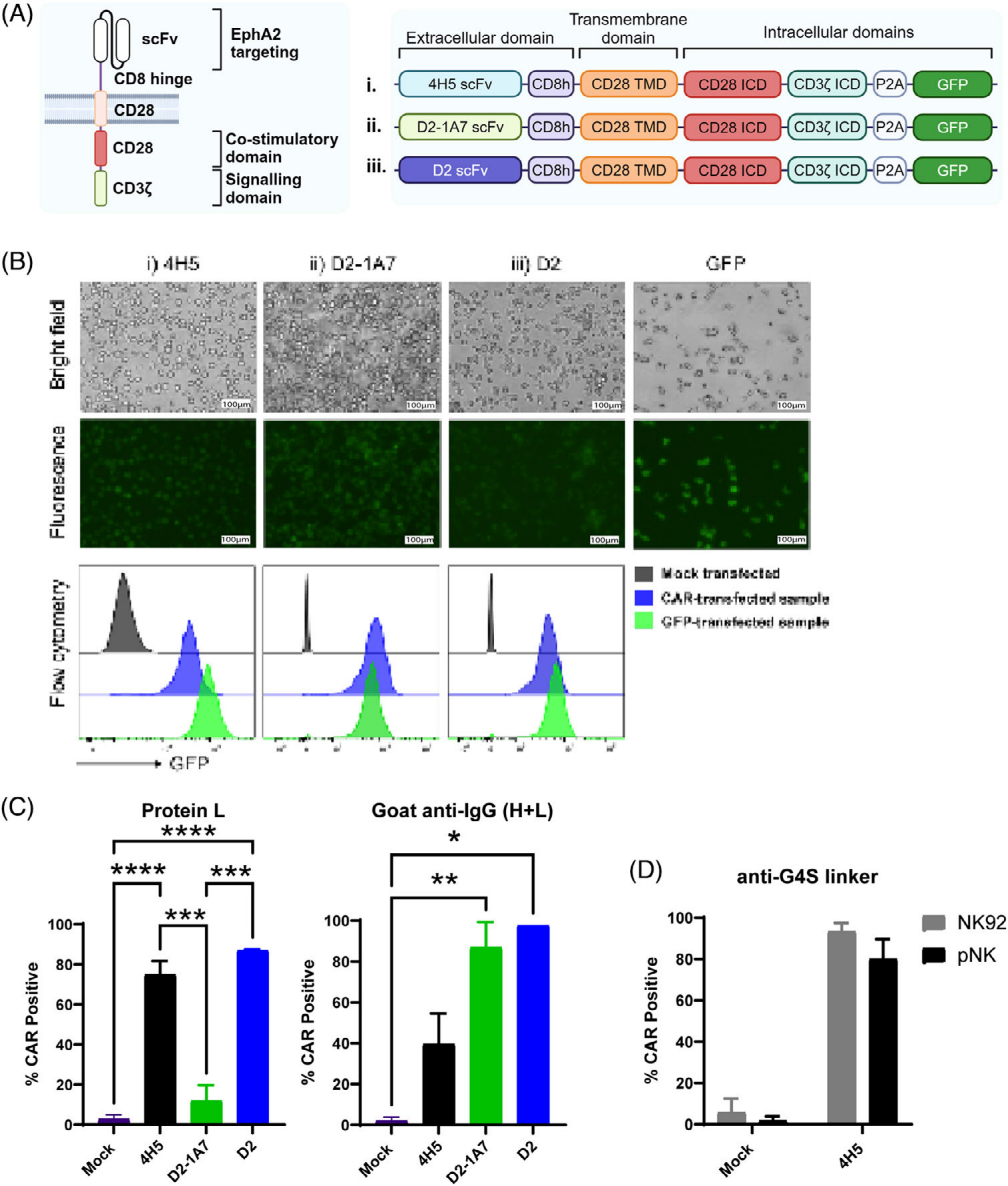
Incorporation of ephrin type‐A receptor‐2 (EphA2)‐targeting clones into a second‐generation chimeric antigen receptor (CAR) design and CAR detection in natural killer (NK) cells. (A) Schematic diagram of three EphA2‐CAR designs derived from three reported anti‐EphA2 antibody clones (4H5, D2 and D2‐1A7). Figure made in BioRender—biorender.com. (B) Expression of GFP observed under fluorescent microscopy or detected using flow cytometry in Khyg1 NK cells mock, EphA2‐CAR or eGFP mRNA transfected 24‐h post‐transfection. (C and D) Various CAR detection methods were tested using flow cytometry including using Protein L (biotinylated or PE‐conjugated), goat anti‐IgG (H+L)‐PE or anti‐G4S linker 24–48 h post‐transfection in (C) Khyg1 cells or in (D) NK92 or human NK cells. Data are presented as mean ± SE and data in (B and C) is pooled from four to six independent experiments while data in (D) are pooled from six to seven independent experiments. Statistical analyses in (C) are carried out using one‐way analysis of variance (ANOVA) (multiple comparisons), where ns: not significant; ^*^ *p *< .05; ^**^ *p *< .01; ^***^ *p *< .001; ^****^ *p *< .0001.

Numerous studies have highlighted EphA2's role in tumour cell proliferation, migration and survival, making it a prime candidate for targeted therapy in solid tumours. The development of anti‐EphA2 humanised antibodies has led to the generation of multiple antibody clones, each designed with slightly different mode of action.^74^ From the array of available clones, we selected three specific anti‐EphA2 clones—4H5, D2 and D2‐1A7 due to availability of sequences in literature. Notably, 4H5 and D2‐1A7 clones were also previously demonstrated to have high specificity against EphA2 in preclinical studies utilising CAR‐T cells^42^, ^46^, ^75^ and antibody‒drug conjugates.^51^ We designed a second‐generation CAR sequence consisting of an scFv sequence derived from the clones selected, linked with a flexible CD8 hinge and CD28 and CD3ζ intracellular signaling domains, followed by a P2A sequence and a GFP sequence (EphA2‐CAR‐GFP) (Figure 2A). To test the delivery of mRNA into NK cells, we generated mRNA of the EphA2‐CAR‐GFP sequence using IVT and transfected it into an NK cell line Khgy1 using the MaxCyte STx super‐electroporator equipment and its respective processing assemblies. EphA2‐CAR‐GFP mRNA generated from all three clones of 4H5, D2 and D2‐1A7 could all be successfully expressed in Khyg1 NK cells (Figure 2B). To validate whether each of the CAR clones can be expressed on the cell surface, we tested various commonly used CAR detection methods. Using Protein L, goat anti‐IgG (H+L) and the newly available anti‐G4S linker antibodies and flow cytometry, we observed different sensitivities of each CAR detection method for the various clones when expressed in Khyg1 cells (Figure 2C) or in NK92 and primary human NK cells (Figure 2D). In our hands, we further observed varying levels of non‐specific staining of mock transfected primary human NK cells using protein L and goat anti‐IgG (H+L) antibody (data not shown). In contrast, little to no non‐specific staining were observed in primary human NK cells using the anti‐G4S linker detection antibody by flow cytometry. We also studied different mRNA deliveries by comparing the MaxCyte STx‐based electroporation with mRNA‐LNP delivery (Figure S1). While the LNP approach effectively delivered mRNA and facilitated CAR expression, cells transfected using the MaxCyte STx system demonstrated significantly higher expression levels. Consequently, this method was selected for the subsequent experiments in this study.

### 3.3 EphA2‐targeting CAR‐NK cells effectively target sarcoma cells in vitro

**FIGURE 3:**
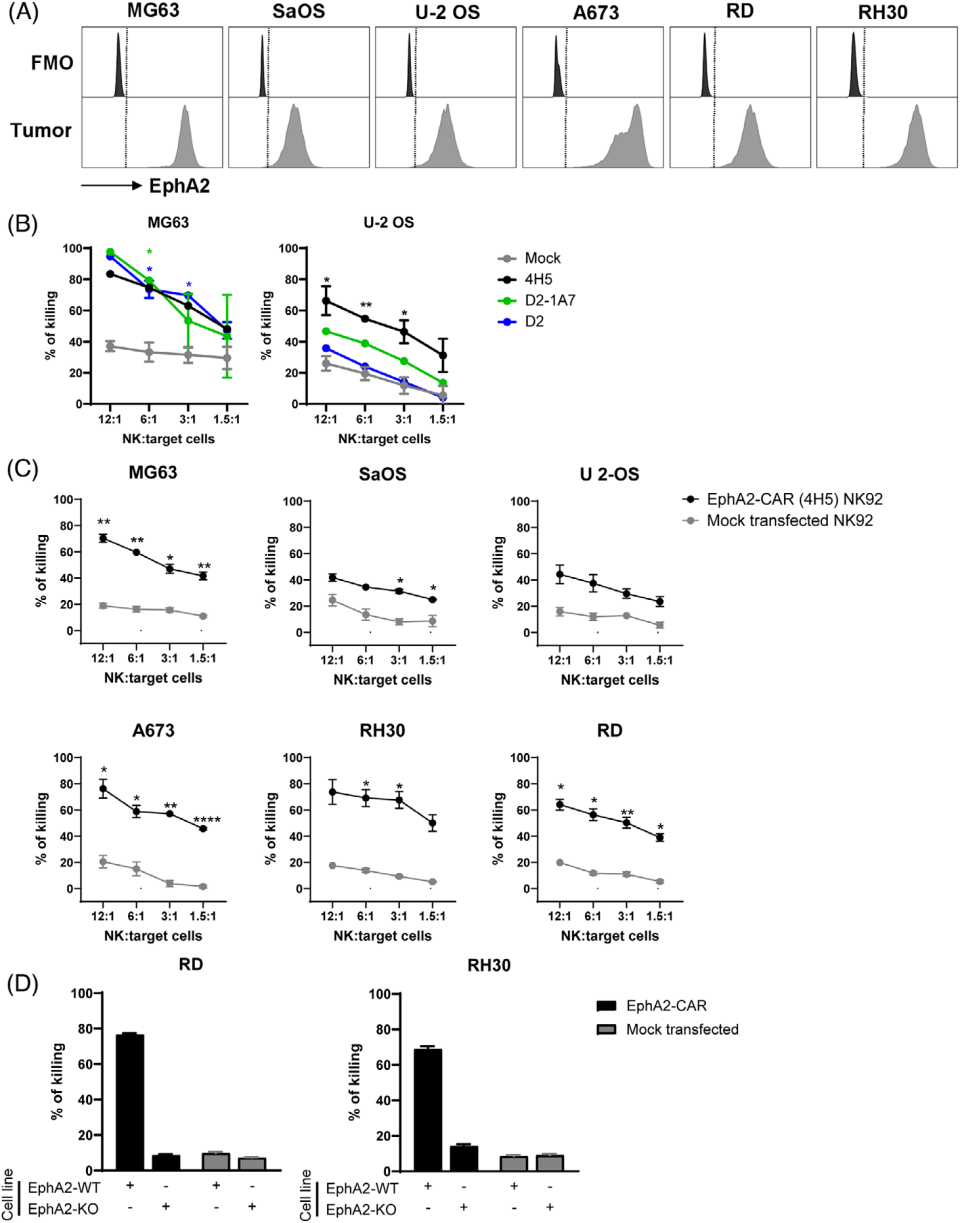
Paediatric sarcomas can be effectively targeted by ephrin type‐A receptor‐2 (EphA2) (4H5)‐chimeric antigen receptor (CAR) natural killer (NK) cells. (A) Cell surface expression of EphA2 was validated in various paediatric osteosarcoma (MG63, SaOS and U2OS), Ewing sarcoma (A673) and rhabdomyosarcoma (RD, RH30) using flow cytometry. Differences in killing abilities of the different clones of EphA2‐CAR in NK92 cells were tested against MG63 and U2OS cell lines (B) using a Calcein AM cytotoxicity assay.^56^, ^57^, ^58^ EphA2 (4H5)‐CAR NK92 cells were further tested against EphA2‐expressing paediatric sarcoma cell lines (SaOS, A673, RD and RH30) to validate the enhancement in killing activity in vitro (C). Killing efficiency was confirmed to be dependent of EphA2 expression in rhabdomyosarcoma (RMS) cells, using EphA2‐KO cells as control (D). Data are representative of two independent experiments, presented as mean ± SE, and statistical analyses compared using unpaired *t*‐test (Holm‒Sidak method) at each effector‐to‐target (E:T) ratio, where ns: not significant; ^*^ *p *< .05; ^**^ *p *< .01; ^****^ *p *< .0001.

We validated EphA2 expression in sarcoma cell lines available in our laboratory (Figure 3A) and tested EphA2‐CAR‐NK cells against these cell lines using a Calcein AM cytotoxicity assay. Cytotoxicity assays were carried out using NK92 cells due to their increased functional activity and clinical relevance as compared to Khyg1 cells (Figure S2A). A comparison of the different clones used to generate EphA2‐CAR mRNA showed that while all three EphA2‐CAR constructs expressed in NK92 cells were highly sensitive against the OS cell line MG63 (Figure 3B), only EphA2‐CAR sequence generated from the 4H5 clone could significantly enhance killing activity of NK92 cells against another OS cell line, U‐2 OS (Figure 3B). These observations indicate that among the three clones tested, EphA2‐CAR generated from the scFv of the 4H5 antibody may have the highest specificity against EphA2 expressed in OS cells. To further validate the efficacy of the EphA2‐CAR generated from the 4H5 clone against other sarcoma cell lines, we transfected NK92 cells with EphA2‐CAR mRNA at a lower and MaxCyte‐recommended concentration of 200 µg/mL, as we were observing higher incidences of cell death after electroporation with 15 pmol of mRNA delivered per million NK cells (data not shown). Here, we observed that EphA2‐CAR‐NK92 cells retained enhanced specificity against OS cell lines MG63 and U‐2 OS, as well as SaOS (Figure 3C). EphA2‐CAR‐NK92 cells also had enhanced killing activity against an EWS cell line A673 and two RMS cell lines, RD and RH30 as compared to mock transfected NK92 cells in vitro. The specificity of the EphA2‐CAR‐NK cells was validated in EphA2‐knockout (KO) RD and RH30 cell lines, where little killing of EphA2‐KO cells was observed (Figure 3D). We further show that EphA2‐CAR could also be effectively delivered into freshly isolated PB‐derived primary human NK cells, which enhanced their killing specificities against EphA2‐positive RD and RH30 cell lines in vitro (Figure S2B). In summary, these results indicate the potential of developing an EphA2‐CAR mRNA sequence based on the 4H5 clone for therapeutic use against sarcoma.

### 3.4 Chemical modifications to mRNA enhance expression of EphA2‐CAR in NK cells

**FIGURE 4:**
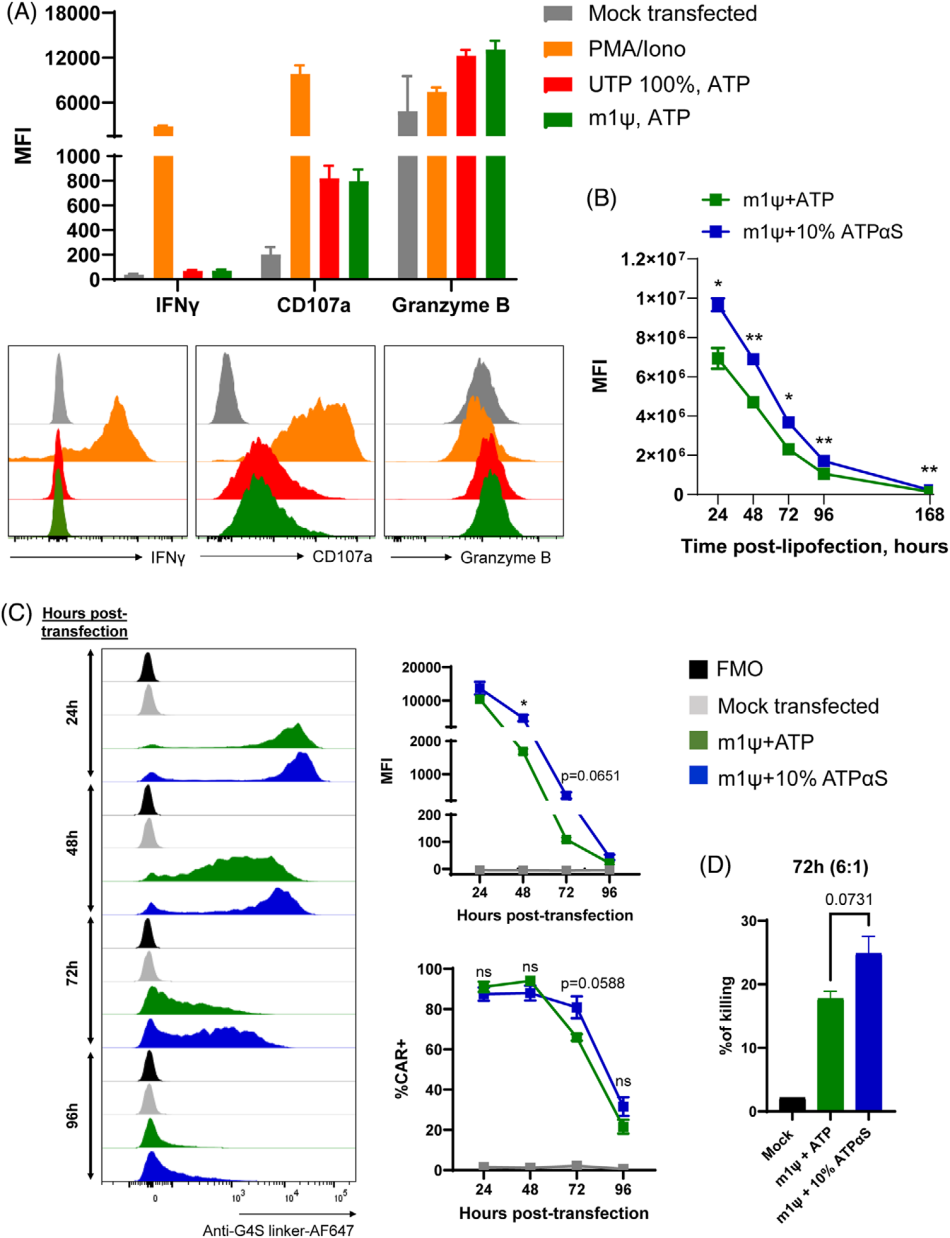
Modifications of mRNA for transient transfection can enhance stability of chimeric antigen receptor (CAR) expression in natural killer (NK) cells. (A) Modification of eGFP mRNA (UTP, 100%) with N^1^‐methylpseudouridine (m1ψ) transfected into primary NK cells does not lead to NK cell activation in vitro. NK cells were assessed for interferon‐gamma (IFN‐γ) and granzyme B production and CD107a expression 5 h post‐transfection with 15 pmol of mRNA/million cells of mRNA. (B) Validation of further modification of m1ψ‐modified eGFP mRNA with 90% ATP + 10% adenosine‐5′‐(α‐thio)‐triphosphate (ATPαS) in HEK293F cells in further enhancing mRNA expression over a period of 168 h by flow cytometry in vitro. (C) Validation of further enhancement of expression of ephrin type‐A receptor‐2 (EphA2)‐CAR mRNA in NK92 cells over a period of 96 h by flow cytometry when transfected with m1ψ‐modified mRNA or m1ψ‐modified + mRNA90% ATP + 10% ATPαS or mock transfected. CAR detection was carried out using 1:50 dilution of anti‐G4S linker antibody. (D) Comparison of EphA2‐CAR NK92 cell killing activity at 72‐h timepoint against RD cell line at the 6:1 (NK cells:target) ratio. Data in (A) are representative of two independent donors from one independent experiment; data in (B and D) are representative of *n* = 3 biological replicates in one independent experiments; data in (C) are representative of *n* = 3 biological replicates from two independent experiments. Data are presented as mean ± SEM and statistical analyses in (B‒D) are carried out using unpaired *t*‐test at each timepoint (Holm–Sidak method), where ns: not significant; ^*^ *p *< .05; ^**^ *p *< .01.

The accelerated development of a COVID‐19 vaccine has allowed for mRNA technologies to progress at unprecedented speed and has shed light on the various potential modifications that could be made at the mRNA level to greatly enhance mRNA stability and reduce innate immunogenicity for therapeutic uses. This was particularly relevant in both COVID‐19 vaccines produced by BioNTech and Moderna,^76^ of which efficacy were shown to be attributed to the incorporation of modified ribonucleotide pseudouridine (Ψ) or N^1^‐pseudomethyluridine (m1Ψ). We have validated the efficacy of delivering and generating EphA2‐CAR mRNA with m1ψ modification to NK92 and primary human NK cells, demonstrating enhanced target specificity against the EphA2 antigen in sarcoma cells. In Figure 4A, we confirm that the use of m1ψ‐modified mRNA (green) does not induce increased immunogenic responses in primary NK cells, as indicated by the comparable levels of key activation markers (granzyme B, CD107a and IFN‐γ) between m1ψ mRNA‐treated cells and control conditions (UTP 100%, ATP; red). The flow cytometry profiles appear similar and quantitative analysis revealed no statistically significant differences between the conditions (*p* > .05), indicating that the m1ψ‐modified mRNA does not elicit an exaggerated immune activation response (Figure 4A). Additionally, this m1ψ modification further enhances protein expression in vitro, as compared to IVT protocols incorporating uridine (data not shown), confirming the suitability of modified mRNA for electroporation into NK cells for transient CAR engineering.

We also tested whether other chemical modifications could be applied to uridine or m1ψ‐modified mRNA for further enhancement of stability of EphA2‐targeting CAR‐NK cells, such as the incorporation of ATPαS in the IVT reaction which introduces phosphorothioate bonds into the mRNA and increases protein yield and reduces RNase‐induced degradation.^77^, ^78^ We first tested the stability of different mRNA formulations such as mRNA chemical compositions incorporating normal uridine, methoxyuridine, m1Ψ, as well as m1Ψ with varying concentrations of ATPαS. All mRNA produced with chemical modifications were found to be stable when stored either at 4°C or RT by achieving satisfactory RNA Integrity Number and concentrations at all conditions (Figure S4). As a starting point, eGFP m1Ψ mRNA were first synthesised containing either 0% or 10% ATPαS. Our results demonstrate that incorporation of 10% ATPαS into eGFP m1Ψ mRNA significantly increases eGFP intensity across 168 h post‐lipofection in HEK293F cells in vitro (Figure 4B). We then synthesised EphA2‐CAR m1ψ mRNA containing 0% or 10% ATPαS and demonstrate a similar trend to eGFP in HEK293F cells across 96 h post‐transfection in NK92 cells (Figures 4C and S3), indicating increased mRNA stability and therefore enhanced and prolonged protein expression with the incorporation of 10% ATPαS. We then extended our comparisons of EphA2‐CAR mRNA transfection (Figure S5) across different sources of clinically relevant NK cells,^79^, ^80^ including PB‐sorted NK cells, the NK92 cell line, and CB‐derived NK cells. These comparisons utilised MaxCyte STx‐based electroporation with various mRNA chemical compositions incorporating the following: normal uridine, methoxyuridine, m1Ψ, as well as m1Ψ with 10%, 25%, or 50% ATPαS. For comparison, LNP‐based transfection using normal uridine mRNA was also tested. The LNP approach consistently demonstrated lower transfection efficiency compared to MaxCyte STx‐based electroporation in PB and CB NK cells. Notably, LNPs failed to transfect NK92 cells, whereas all tested chemical modifications and normal uridine mRNA achieved efficient EphA2‐CAR expression in NK cells using MaxCyte STx (Figure S5A). Furthermore, cytotoxicity assays were performed on PB and CB NK cells, as well as NK92 cells, following transfection. These assays revealed no significant differences in the enhancement of killing capacity against the target sarcoma cell line between the various mRNA chemical modifications (Figure S5B) in each cell type. In a time point‐kinetic experiment, we observed optimal CAR expression after transfection with m1Ψ mRNA at the 24‐h mark (Figure S6). Notably, NK92 cells equipped with EphA2‐CAR translated using m1Ψ + 10% ATPαS had higher killing activity against RD cells, albeit not statistically significant, as compared to EphA2‐CAR‐NK92 cells generated using m1Ψ mRNA 72 h post‐electroporation (Figure 4D). These observations indicate that ATPαS modifications, among other possible modifications, are suitable to generate more stable expression of CARs in NK cells for future therapeutic purposes against sarcoma.

### 3.5 EphA2‐CAR‐NK cells exhibit enhanced anti‐tumour activity in sarcoma‐bearing mouse models

**FIGURE 5:**
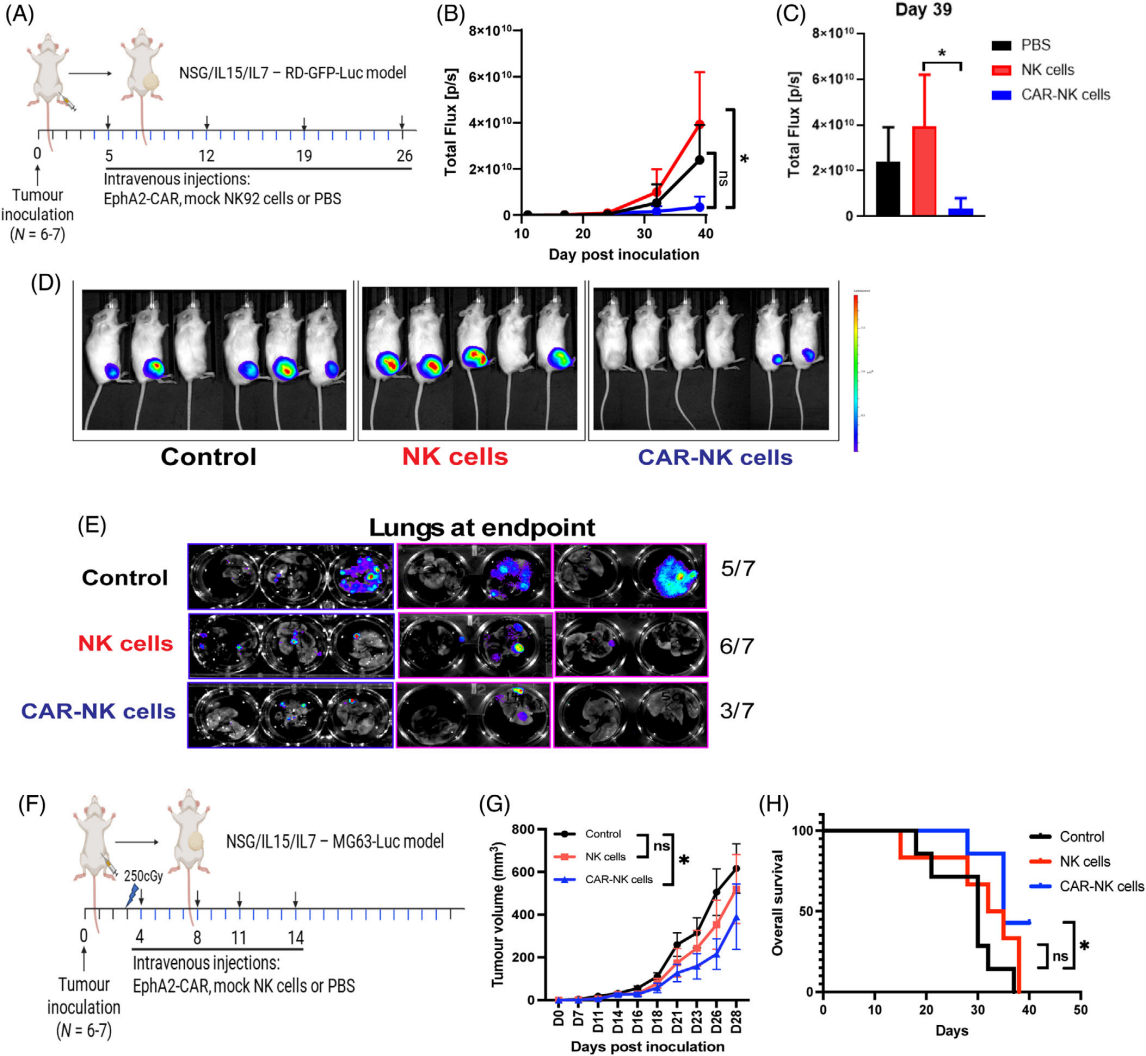
Ephrin type‐A receptor‐2 (EphA2)‐chimeric antigen receptor (CAR) natural killer (NK) cells anti‐tumour activity in sarcoma‐bearing mice. (A) Dose schedule diagram of the RD in vivo experiment, where GFP^+^ Luc^+^ RD cells were orthotopically inoculated in NSG/A2/hIL‐7/hIL‐15‐KI mice, followed by treatment four weekly doses of CAR‐NK92, mock transfected NK92 cells or left untreated (PBS or control). Figure made in BioRender—biorender.com. (B) Total flux detection from luciferase/luciferin reaction in RD‐bearing mice by IVIS over time. (C) Total flux detection from luciferase/luciferin reaction in RD‐bearing mice by IVIS over time at day 39. (D) Representative IVIS images in RD‐bearing mice at day 39. (E) Mice treated with CAR‐PB‐NK cells, PB‐NK cells or untreated were sacrificed and necropsied on day 42 post‐inoculation. Ex vivo IVIS images demonstrate bioluminescent signal detected in the lungs indicating metastasis and ratio of metastatic‐positive lungs per group is indicated on numbers on right. (F) Dose schedule diagram of the MG63 GFP^+^ Luc^+^ subcutaneous model in NSG/A2/hIL‐7/hIL‐15‐KI mice, followed by treatment with four doses of CAR‐NK cells (PB‐derived), mock NK cells or left untreated (PBS or control). Figure made in BioRender—biorender.com. (G) Primary MG63 tumour growth over time was measured. (H) Mice displaying disease score closer to the ethical threshold were culled, and overall survival was calculated and compared between groups. Statistical analyses between multiple groups in (A) were carried out using Kruskal‒Wallis test, in (B) by unpaired *t*‐test, in (G) by two‐way analysis of variance (ANOVA) with Tukey's multiple comparison test and in (H) by Gehan‒Breslow‒Wilcoxon test, where ^*^ *p *< .05. Results were generated from *n* = 6‒7 mice per group per experiment.

Finally, we evaluated the anti‐tumour efficacy of EphA2‐CAR‐NK cells in distinct in vivo sarcoma models. In an RMS model, where RD cells were orthotopically inoculated into mice (Figure 5A), i.v. weekly administration of EphA2‐CAR‐NK92 or fortnightly pNK cells significantly suppressed local tumour progression (Figure 5B‒D). In the same setting, a subsequent experiment was performed to analyse metastatic burden in the lungs, which was observed to be reduced in mice treated with EphA2‐CAR‐NK cells (Figure 5E). In a separate OS model using MG63 cells (s.c. inoculated) and PB‐derived EphA2‐CAR‐NK cells for treatment (Figure 5F), primary tumour growth was markedly inhibited in the EphA2‐CAR‐NK cell group (Figure 5G), accompanied by a notable survival benefit (Figure 5H). These findings demonstrate that EphA2‐CAR expression, in both NK92 cells and primary blood‐derived NK cells, enhances their anti‐tumour activity and survival outcomes in different sarcoma models.

## 4 DISCUSSION

CAR‐T cell therapies have transformed the field of cancer immunotherapy, leading to improved immune responses against cancer. Even though the application of CAR‐T cells has drawbacks, CARs are proving invaluable in the field of immunotherapy by providing a highly specific and adaptable platform for targeting cancer cells. This adaptability has facilitated the development of CAR constructs for various immune cells beyond T cells, including NK cells, thereby expanding the therapeutic potential of CAR‐based treatments. As a result, ongoing research is focused on optimising CAR constructs, minimising off‐target effects, improving the overall safety profile of CAR therapies and opening new horizons with enormous potential to explore mRNA‐based expression systems for CAR immunotherapeutics.^81^ This report details the design and validation of EphA2‐targeting CAR‐NK cells for use against paediatric sarcomas using mRNA‐based transfection systems to transiently engineer hard‐to‐edit NK cells for therapeutic purposes. Furthermore, we show that specific chemical modifications can enhance stability of mRNA for transient engineering, leading to enhanced stability and therefore protein expression of EphA2‐CAR in NK cells in vitro. These observations expand on the feasibility and applicability of mRNA‐based transient engineering of NK cells as a therapeutic modality.

Our study leveraged data from the TARGET‐OS program (accession study phs000468.v18.p7) and the E‐TABM‐1202 dataset for RMS^59^ to evaluate the prognostic impact of NK cell infiltration and EphA2 expression. The analysis demonstrated a positive correlation between higher NK cell scores and improved 5‐year survival rates in OS patients, and inversely, a positive correlation between higher EphA2 expression scores with poorer 5‐year survival rates in OS and RMS patients. Our robust NK cell scoring system^62^ reinforces the observation that they are a crucial component in the anti‐tumour immune response and significantly impact patient outcomes.^82^ EphA2 has emerged as a significant target in various solid tumours, as its role in tumour angiogenesis, cell migration, and survival contributes to its oncogenic potential^74^ and many studies have explored the targeting of EphA2‐CAR T cells against a variety of solid tumours, including preclinical models of OS and EWS.^83^, ^84^ Given the strong association between NK cell infiltration in solid tumours and overall patient survival^82^ and their favourable safety profile compared to T cells,^19^, ^21^, ^26^ combining these properties in anti‐cancer therapies is a logical and promising strategy.

In considering the clinical translation of CAR‐NK cell therapies, it is critical to address safety aspects, particularly regarding off‐target effects and immunogenicity. EphA2, while significantly overexpressed in sarcomas, is also present in some healthy tissues, including highly proliferating normal epithelial cells,^38^ posing a theoretical risk of unintended targeting. Importantly, clinical trials to date have not indicated off‐target effects, likely due to the capacity of NK cells to recognise and spare healthy cells through inhibitory signals from MHC class I/HLA expression.^25^ Here, we have selected EphA2 as a target for proof‐of‐concept purposes; however, identifying more selective markers that are minimally expressed in normal paediatric tissues remains an active area of investigation to enhance the safety of CAR‐NK cell therapies for sarcoma.

CARs have evolved through four generations, each improving upon the previous to enhance immune cell function and efficacy.^85^ In this study, we utilised a second‐generation CAR design that uses CD28 and CD3ξ intracellular domains that have been reported for use in approved CAR‐T cell treatments in clinical trials^86^ and in early CAR‐NK cells studies^87^ as a reference starting point in designing an EphA2‐CAR sequence. While the rationale for the initial incorporation of CD28 as a co‐stimulatory molecule in a second‐generation CAR for NK cells was less well‐defined,^88^ it has recently been validated to result in enhanced CAR‐NK cell persistence and sustained anti‐tumour cytotoxicity.^89^ Likewise, a second generation EphA2‐CAR incorporating a CD8a transmembrane, and CD137, and CD3z costimulatory domains virally transduced into memory‐like NK cells at 20%–30% CAR positivity could also significantly enhance NK cell activity against preclinical models of HNSCC.^47^ Our results comparing the different clones show that the scFv derived from the 4H5 clone of anti‐human EphA2 antibody, which is a humanised version of the EA2 clone of the anti‐mouse EphA2 antibody,^49^ was most effective for the recognition and killing of sarcoma tumour cells by EphA2‐CAR‐NK cells in vitro. Indeed, the EA2 clone has been previously shown to stably bind the EphA2 protein epitope in a conformation found only in malignant cells^90^ and would explain why the 4H5 clone is one of the more commonly explored clones developed for therapeutic purposes. In sum, our results confirm the suitability of using such co‐stimulatory domains as well as the incorporation of the scFv region of the 4H5 clone in both NK cell lines and primary human NK cells in the enhancement of tumour‐targeted cytotoxicity in vitro.

PB‐derived NK cells, CB‐derived NK cells and NK92 cells each present unique advantages and challenges as sources for NK cell‐based therapies.^79^, ^80^ PB‐derived NK cells are particularly promising due to their superior capacity for expansion and genetic engineering compared to CB‐derived NK cells, which require careful coordination with birth clinics to obtain and are therefore less accessible.^80^ In contrast, NK92 cells offer ease of culture and genetic manipulation but are subject to licensing requirements, making their clinical translation more complex. In our study, PB‐derived NK cells demonstrated robust transfection efficiency and cytotoxic performance, highlighting their potential as an economically viable and scalable source for mRNA‐based CAR‐NK cell therapies. These findings support PB‐derived NK cells as a practical and effective choice for advancing this therapeutic approach.

mRNA technologies have revolutionised not only the development of vaccines but are also emerging as a groundbreaking platform for cancer therapies. This advancement is particularly valuable in NK cell therapies, where traditional genetic editing techniques often face challenges due to the inherent difficulty of modifying NK cells. Notably, success rates of successfully virally transducing primary human NK cells with CAR sequences can reportedly range widely from 19% to 73%,^91^ whereas we and others show that transient electroporation of CAR sequences in NK cells can consistently reach more than 80% one day after electroporation.^92^, ^93^ However, this emerging platform is not without drawbacks as the transient nature of mRNA expression often requires repeated administrations of CAR‐NK cells to maintain therapeutic effects. Nevertheless, various studies have demonstrated that mRNA‐edited NK cell therapies possess significant therapeutic potential and should not be dismissed despite their challenges.^81^ The transient nature of mRNA expression offers the advantage of controlled, on‐demand modulation of NK cell functions, allowing for flexible and adjustable treatment regimens. Moreover, advancements in mRNA stabilisation and delivery techniques are continually improving the durability and efficiency of mRNA‐based modifications. Here, we show that current mRNA technologies incorporating m1Ψ can be enhanced through further chemical modifications using 10% hydrolysis‐resistant ATP analogue, ATPαS, as an optimal concentration that does not compromise mRNA yield (data not shown) or performance.^77^ Either too low or too high ATPαS concentrations can impede ribosomal sliding and tRNA recognition due to its unusual chemical properties.^77^, ^78^ ATPαS is presumed to prevent or delay mRNA degradation from the 3′ end, particularly by stabilising the PolyA tail, thus enhancing mRNA stability and prolonging its expression.^77^ In *Escherichia coli*, ATPαS increases scanning ribosome number, suggesting improved translation initiation in ATPαS‐containing mRNAs. Thus, our results indicate that ATPαS could have induced a similar effect on eukaryotic, that is, NK cell translation complexes. To our knowledge, this is the first study reporting on the testing of modified mRNA incorporating m1Ψ and ATPαS modifications to improve the stability of CAR protein expression. Future studies should explore the optimisation of other chemical modifications, as different modifications may exhibit varying levels of effectiveness depending on the specific mRNA application. While our in vitro data indicate that CAR expression and killing capacity decline over time post‐transfection, it is important to note that in vivo, the rate of mRNA dilution may differ significantly. In a clinical setting, infused NK cells are unlikely to proliferate, and dilute mRNA as rapidly as observed in culture, suggesting that therapeutic effects may persist longer than our in vitro findings suggest. These dynamics, however, warrant further investigation in appropriate in vivo models evaluating their impact on NK cell viability and function over extended periods, as well as therapeutic efficacy to harness their full potential for cancer immunotherapy.

In summary, CAR‐T cell therapies have revolutionised cancer treatment by providing targeted and adaptable approaches to enhance immune responses against cancer. This study extends this innovation to NK cells by developing and validating EphA2‐targeting CAR‐NK cells through mRNA‐based transfection, showcasing improved mRNA stability via specific chemical modifications. Our findings underscore the importance of NK cells in anti‐tumour immunity and highlight EphA2 as a critical oncotarget, with higher NK cell scores correlating with better survival in OS patients. Our research contributes to the broadening of the scope of CAR technology in NK cells, reinforces the potential of mRNA engineering to overcome the challenges of modifying NK cells and offers promising prospects for developing more effective and versatile cancer therapies. Advancing EphA2‐CAR‐NK cell therapies toward clinical application will require further in vivo preclinical studies using advanced animal models that better mimic the human tumour microenvironment and its immunosuppressive challenges. Combining EphA2‐CAR‐NK cells with immune checkpoint inhibitors or other immune‐modulating agents may enhance therapeutic efficacy and durability. Additionally, refining CAR specificity and identifying highly tumour‐selective targets will be essential to minimise risks to healthy tissues and improve safety. Addressing these aspects represents a crucial step in moving CAR‐NK cell therapies from preclinical models to potential clinical use, broadening the scope of NK cell‐based immunotherapy for paediatric sarcomas.

## AUTHOR CONTRIBUTIONS

*Conception and design of the study*: Pui Yeng Lam, Natacha Omer, Wayne Nicholls, Seth W. Cheetham and Fernando Souza‐Fonseca‐Guimaraes. *Acquisition of data*: Pui Yeng Lam, Gustavo R. Rossi, Hannah Tompkins, Cui Tu, Natacha Omer, Josh K.M. Wong, Jane Sun, Maria Victorova, Elaina Coleborn, Cheng‐Yu Lin, Ahmed M. Mehdi, Amos Choo, Zewen Kelvin Tuong, Louisa Alim and Fernando Souza‐Fonseca‐Guimaraes. *Development of critical reagents*: Lachlan J. Dobson, Alexander D. McLellan and Timothy Mercer. *Analysis and interpretation of data*: Pui Yeng Lam, Cui Tu, Natacha Omer, Josh K.M. Wong, Gustavo R. Rossi, Maria Victorova, Hannah Tompkins, Cheng‐Yu Lin, Ahmed M. Mehdi, Andrew Brooks, Seth W. Cheetham, Amos Choo, Zewen Kelvin Tuong and Fernando Souza‐Fonseca‐Guimaraes. *Drafting the manuscript*: Pui Yeng Lam, Cui Tu, Natacha Omer, Seth W. Cheetham, Wayne Nicholls and Fernando Souza‐Fonseca‐Guimaraes. All authors have revisited and approved the final article.

## CONFLICT OF INTEREST STATEMENT

F.S.F.G is a Board Member of Cure Cancer Australia Foundation and member of the Scientific Advisory Committee of ANZSA. Microba Life Sciences sponsors research in the laboratory of F.S.F.G. Other authors have no commercial, proprietary or financial interest in this study.

## ETHICS STATEMENT

All experiments conducted in this study used human‐derived materials in compliance with ethical guidelines and were approved by the Human Research Ethics Committee (HREC) at the University of Queensland (approval number: 2023/HE000027) and Metro South Health (approval number: HREC/2019/QMS/55385, ratified by the UQ HREC). All animal experiments were conducted following the animal ethics guidelines provided by the National Health and Medical Research Council of Australia and approved by the University of Queensland—Health Sciences Animal Ethics Committee (ethics number: 2021/AE000294).

## Supporting information




**FIGURE S1**. Transfection efficiency of lipid nanoparticle (LNP)‐based or MaxCyte STx electroporation for chimeric antigen receptor (CAR) mRNA delivery in natural killer (NK) cells. Primary PB‐derived NK cells were transfected either by LNP particles carrying the ephrin type‐A receptor‐2 (EphA2)‐mRNA or by the MaxCyte STx‐based electroporation. (A) Quantitative comparison of G4S linker staining after the different transfection approaches using three different donors. (B) Representative histogram overlay shows representative staining of the G4S linker. Data are presented as mean ± SEM and statistical analyses in are carried out using unpaired *t*‐test; ^*^ *p *< .05; ^***^ *p *< .001.

**FIGURE S2**. Ephrin type‐A receptor‐2 (EphA2)‐chimeric antigen receptor (CAR) NK92 cells selectively and effectively target EphA2‐expressing cells in vitro. (A) Validation of killing capabilities of NK92 and Khyg1 cells against K562 cells are compared using a Calcein AM killing assay. (B) EphA2 (4H5)‐CAR NK92 cells display high specificity towards EphA2‐expressing rhabdomyosarcoma (RMS) cells in vitro (effector‐to‐target [E:T] ratio 12:1) but not EphA2‐knockout (KO) RMS cell lines. PB‐natural killer (NK) cells were isolated and activated for 24 h with complete media before mock electroporation or with EphA2‐CAR mRNA. Calcein AM cytotoxicity assay was carried out 24 h after electroporation and tested against RMS cell lines. Data are presented as mean ± SE. Data in (A) were pooled from two independent experiments, data in (B) were from one experiment, using *n* = 1 donor from one experiment.

**FIGURE S3**. Representative ephrin type‐A receptor‐2 (EphA2)‐chimeric antigen receptor (CAR) expression in NK92 over time. Overlays are representative of two different mRNA batches encoding EphA2 in a time‐point kinetics of expression in transfected NK92 in technical triplicate.

**FIGURE S4**. Validation of size and stability at 4°C and room temperature of purified mRNA sequence produced by UQ BASE facility using RNA screentape. (A) Electropherogram output from the Agilent ScreenTape assay with corresponding samples in lanes 1–3 showing mRNA integrity for unpurified and purified samples. Distinct bands corresponding to the expected target mRNA sizes are present, indicating successful transcription and purification. Volume in table indicates total volume loaded onto the screentape, including 5.0 µL of RNA loading buffer per 1 µL of diluted sample. (B and C) Stability of the different modified mRNAs was also confirmed after 1 week at 4°C or room temperature (RT). RNA integrity numbers (RIN) and concentrations (conc.) were calculated from the Bioanalyser. SCH_01 = normal uridine; SCH_02 = methoxyuridine; SCH_03 = N1‐methylpseudouridine (m1ψ); SCH_04 = 10% ATP‐5′‐(α‐thio)‐triphosphate (ATPαS); SCH_05 = 25% ATPαS; SCH_06 = 50% ATPαS.

**FIGURE S5**. Comparison of ephrin type‐A receptor‐2 (EphA2)‐chimeric antigen receptor (CAR) mRNA transfection and cytotoxicity in natural killer (NK) cells using different sources, chemical modifications and delivery methods. (A) Transfection efficiency: EphA2‐CAR expression was assessed in PB‐sorted NK cells, NK92 cell lines and cord blood (CB)‐derived NK cells following transfection using MaxCyte STx electroporation with different mRNA modifications (normal uridine, methoxyuridine, N1‐methylpseudouridine (m1ψ) and mRNA incorporating 10%, 25% and 50% ATP‐5′‐(α‐thio)‐triphosphate [ATPαS]). Lipid nanoparticle (LNP)‐based transfection was also tested for comparison. MaxCyte STx achieved significantly higher transfection efficiency across all tested NK cell sources compared to LNPs. LNPs failed to transfect NK92 cells effectively, whereas MaxCyte STx with all tested mRNA modifications achieved robust CAR expression. (B) Cytotoxicity assays: specific lysis of MG32 target cells was evaluated in PB‐NK, NK92 and CB‐NK cells at varying effector‐to‐target (E:T) ratios (6:1, 3:1 and 1.5:1) after transfection with EphA2‐CAR mRNA. Differences in killing capacity were compared between different mRNA modifications, indicating that all tested chemical modifications support comparable cytotoxic activity post‐transfection. Data were analysed using a two‐way analysis of variance (ANOVA) with Tukey's multiple comparisons test. Statistical comparisons are annotated as non‐significant (ns). Data are represented as mean ± SEM.

**FIGURE S6**. Temporal analysis of ephrin type‐A receptor‐2 (EphA2)‐chimeric antigen receptor (CAR) mRNA transfection efficiency and cytotoxic activity in peripheral blood‐derived natural killer (NK) cells. PB‐NK cells transfected with EphA2‐CAR mRNA using the MaxCyte STx platform were evaluated at 24, 48 and 72 h post‐transfection for the percentage of CAR‐expressing cells (A), expression levels measured by mean fluorescence intensity (MFI) (B), and cytotoxicity against MG63 target cells at a 6:1 effector‐to‐target (E:T) ratio (C). Data were analysed using a two‐way analysis of variance (ANOVA) with Tukey's multiple comparisons test. Statistical comparisons are indicated, with non‐significant differences denoted as ‘ns’. Data are presented as mean ± SEM.

## Data Availability

The data that support the findings of this study are openly available in the TARGET‐OS database at https://www.cancer.gov/ccg/research/genome‐sequencing/target/usingtarget‐data (accession study phs000468.v18.p7), ArrayExpress database at https://www.ebi.ac.uk/biostudies/arrayexpress (accession ID E‐TABM‐1202 RMS dataset, Refs.^63^, ^64^ and GSE147390).
