# Supplementary figures and images for: Enhancement of anti‐sarcoma immunity by NK cells engineered with mRNA for expression of a EphA2‐targeted CAR
Source: Clin Transl Med. 2025 Jan 6;15(1):e70140. doi: 10.1002/ctm2.70140 (PMC11705447; doi:10.1002/ctm2.70140)

A

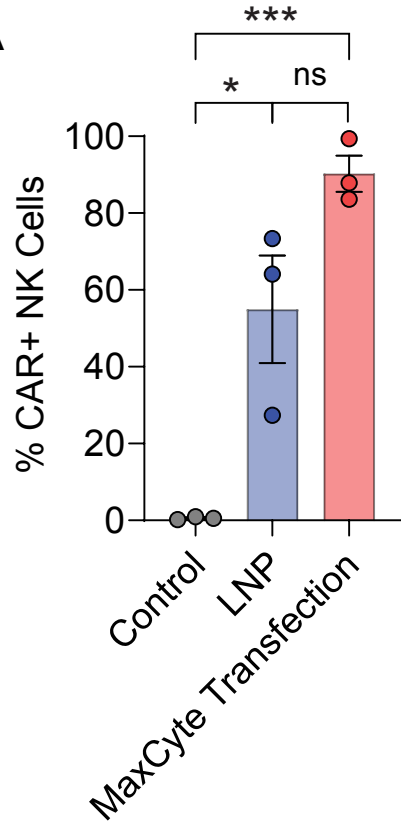

B

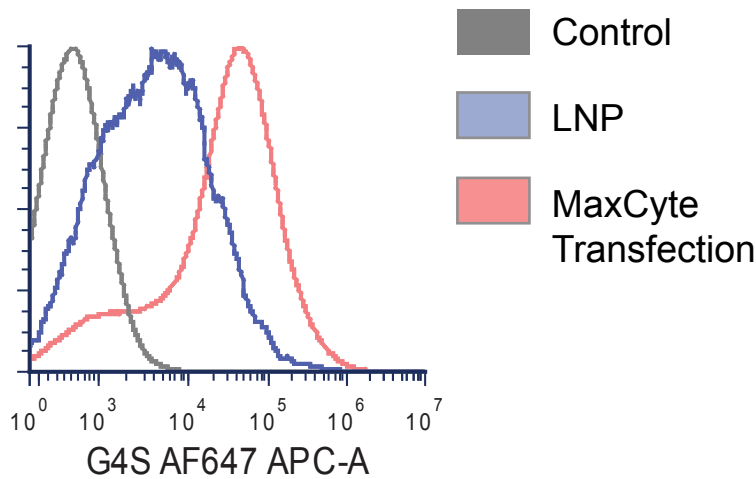

Supplement: Supplementary file 1 — FIGURE S1. Transfection efficiency of lipid nanoparticle (LNP)‐based or MaxCyte STx electroporation for chimeric antigen receptor (CAR) mRNA delivery in natural killer (NK) cells. Primary PB‐derived NK cells were transfected either by LNP particles carrying the ephrin type‐A receptor‐2 (EphA2)‐mRNA or by the MaxCyte STx‐based electroporation. (A) Quantitative comparison of G4S linker staining after the different transfection approaches using three different donors. (B) Representative histogram overlay shows representative staining of the G4S linker. Data are presented as mean ± SEM and statistical analyses in are carried out using unpaired t‐test; * p < .05; *** p < .001. [file CTM2-15-e70140-s003.pdf]

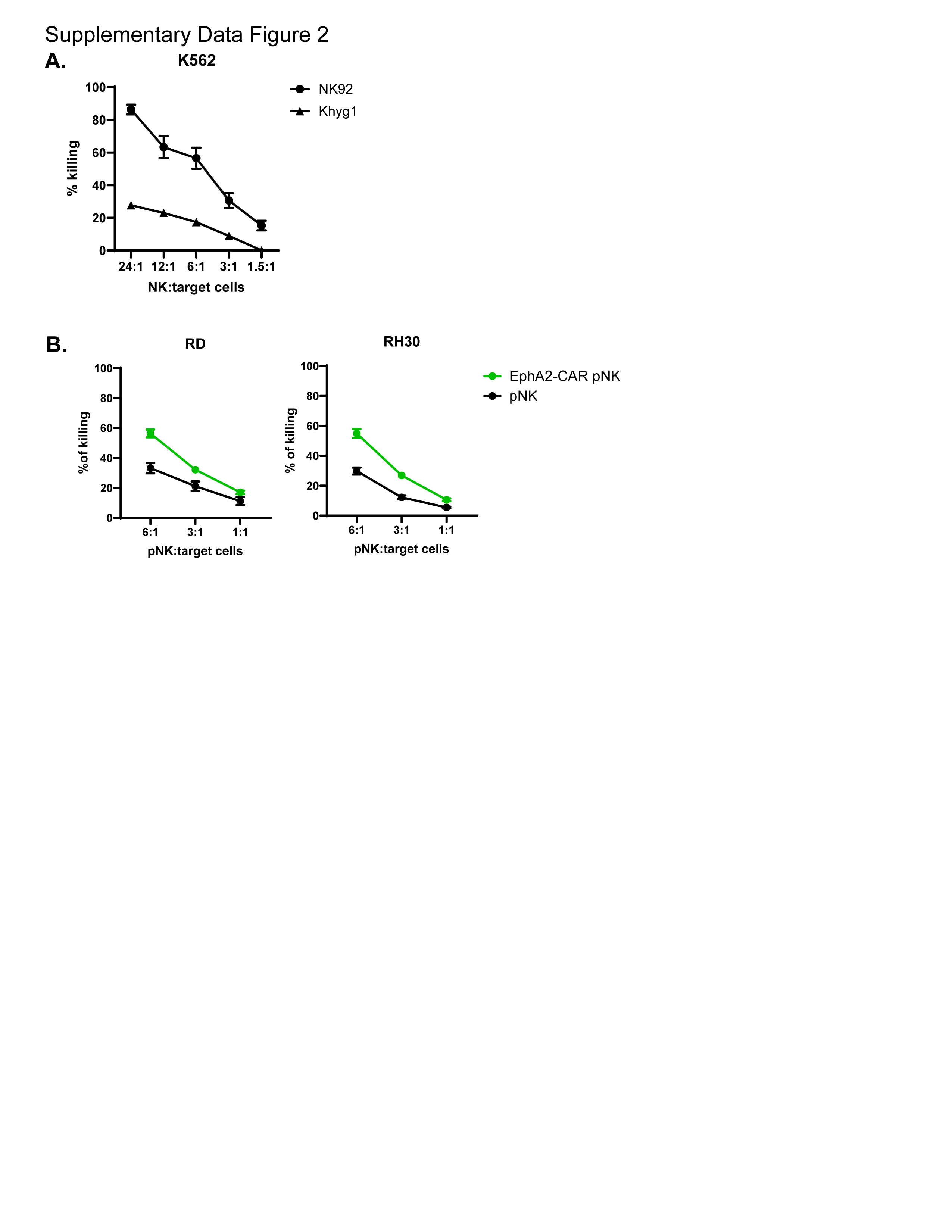

Supplement: Supplementary file 2 — FIGURE S2. Ephrin type‐A receptor‐2 (EphA2)‐chimeric antigen receptor (CAR) NK92 cells selectively and effectively target EphA2‐expressing cells in vitro. (A) Validation of killing capabilities of NK92 and Khyg1 cells against K562 cells are compared using a Calcein AM killing assay. (B) EphA2 (4H5)‐CAR NK92 cells display high specificity towards EphA2‐expressing rhabdomyosarcoma (RMS) cells in vitro (effector‐to‐target [E:T] ratio 12:1) but not EphA2‐knockout (KO) RMS cell lines. PB‐natural killer (NK) cells were isolated and activated for 24 h with complete media before mock electroporation or with EphA2‐CAR mRNA. Calcein AM cytotoxicity assay was carried out 24 h after electroporation and tested against RMS cell lines. Data are presented as mean ± SE. Data in (A) were pooled from two independent experiments, data in (B) were from one experiment, using n = 1 donor from one experiment. [file CTM2-15-e70140-s005.tif]

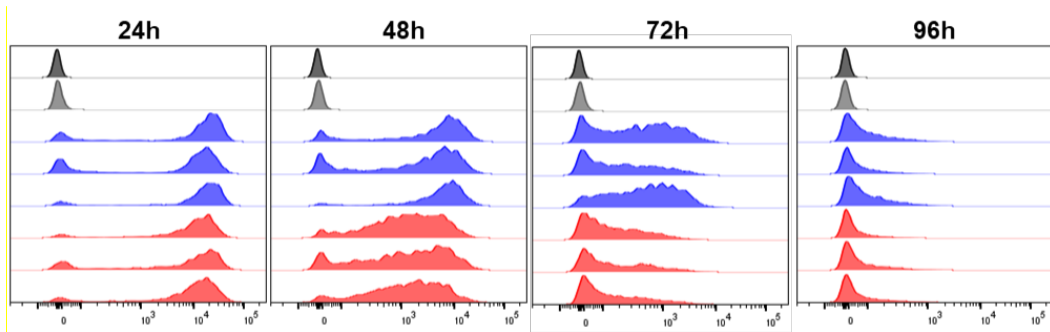

Key: FMO, non-transfected, mRNA-batch 1 (10% ATP $\alpha$ S), mRNA-batch 2 (0% ATP $\alpha$ S)

Supplement: Supplementary file 3 — FIGURE S3. Representative ephrin type‐A receptor‐2 (EphA2)‐chimeric antigen receptor (CAR) expression in NK92 over time. Overlays are representative of two different mRNA batches encoding EphA2 in a time‐point kinetics of expression in transfected NK92 in technical triplicate. [file CTM2-15-e70140-s001.pdf]

**A**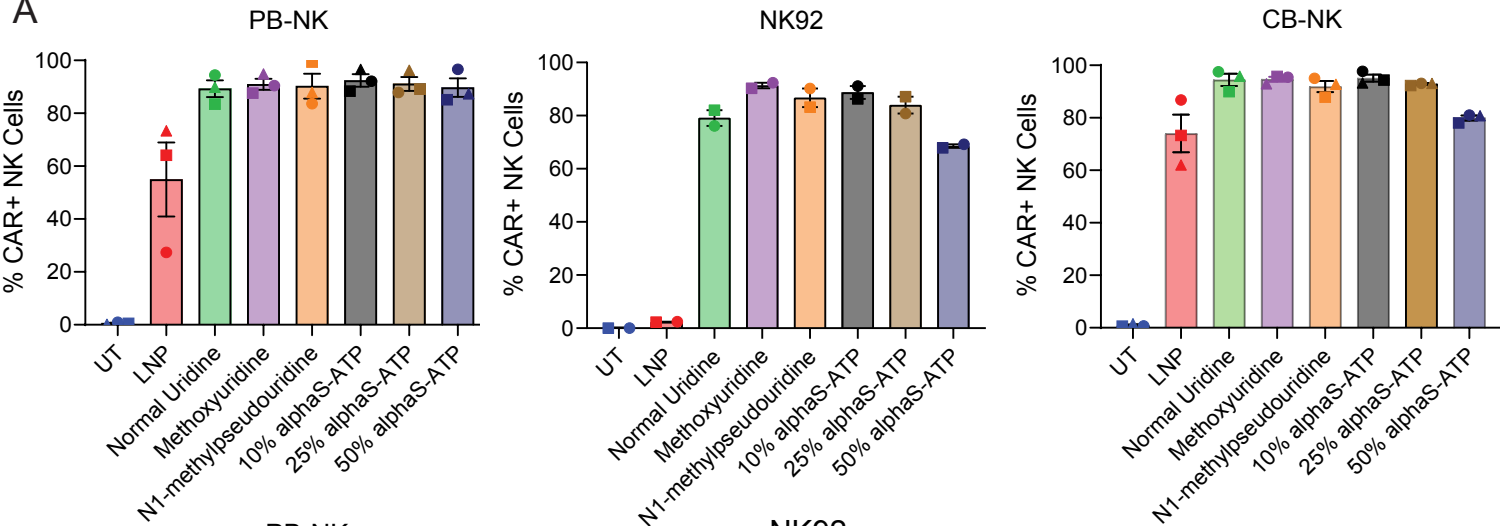**B**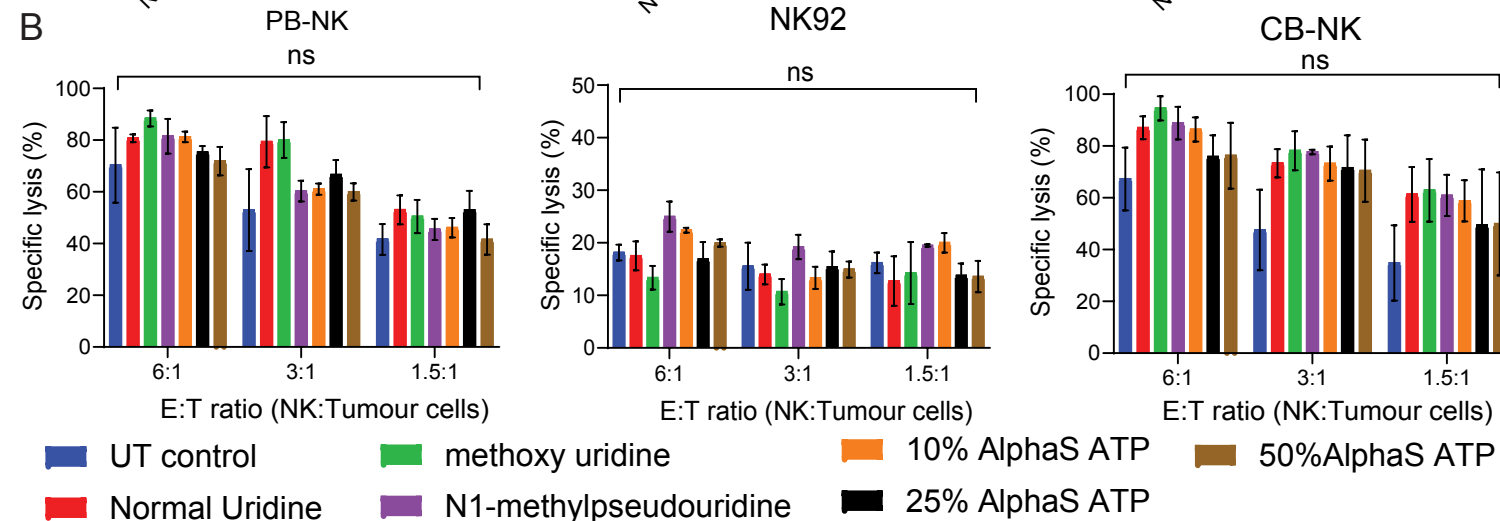

Supplement: Supplementary file 5 — FIGURE S5. Comparison of ephrin type‐A receptor‐2 (EphA2)‐chimeric antigen receptor (CAR) mRNA transfection and cytotoxicity in natural killer (NK) cells using different sources, chemical modifications and delivery methods. (A) Transfection efficiency: EphA2‐CAR expression was assessed in PB‐sorted NK cells, NK92 cell lines and cord blood (CB)‐derived NK cells following transfection using MaxCyte STx electroporation with different mRNA modifications (normal uridine, methoxyuridine, N1‐methylpseudouridine (m1ψ) and mRNA incorporating 10%, 25% and 50% ATP‐5′‐(α‐thio)‐triphosphate [ATPαS]). Lipid nanoparticle (LNP)‐based transfection was also tested for comparison. MaxCyte STx achieved significantly higher transfection efficiency across all tested NK cell sources compared to LNPs. LNPs failed to transfect NK92 cells effectively, whereas MaxCyte STx with all tested mRNA modifications achieved robust CAR expression. (B) Cytotoxicity assays: specific lysis of MG32 target cells was evaluated in PB‐NK, NK92 and CB‐NK cells at varying effector‐to‐target (E:T) ratios (6:1, 3:1 and 1.5:1) after transfection with EphA2‐CAR mRNA. Differences in killing capacity were compared between different mRNA modifications, indicating that all tested chemical modifications support comparable cytotoxic activity post‐transfection. Data were analysed using a two‐way analysis of variance (ANOVA) with Tukey's multiple comparisons test. Statistical comparisons are annotated as non‐significant (ns). Data are represented as mean ± SEM. [file CTM2-15-e70140-s002.pdf]

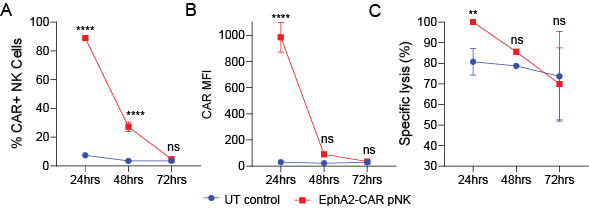

Supplement: Supplementary file 6 — FIGURE S6. Temporal analysis of ephrin type‐A receptor‐2 (EphA2)‐chimeric antigen receptor (CAR) mRNA transfection efficiency and cytotoxic activity in peripheral blood‐derived natural killer (NK) cells. PB‐NK cells transfected with EphA2‐CAR mRNA using the MaxCyte STx platform were evaluated at 24, 48 and 72 h post‐transfection for the percentage of CAR‐expressing cells (A), expression levels measured by mean fluorescence intensity (MFI) (B), and cytotoxicity against MG63 target cells at a 6:1 effector‐to‐target (E:T) ratio (C). Data were analysed using a two‐way analysis of variance (ANOVA) with Tukey's multiple comparisons test. Statistical comparisons are indicated, with non‐significant differences denoted as ‘ns’. Data are presented as mean ± SEM. [file CTM2-15-e70140-s006.png]
