# Supplementary material for: Enhancement of anti‐sarcoma immunity by NK cells engineered with mRNA for expression of a EphA2‐targeted CAR
Source: Clin Transl Med. 2025 Jan 6;15(1):e70140. doi: 10.1002/ctm2.70140 (PMC11705447; doi:10.1002/ctm2.70140)

A.

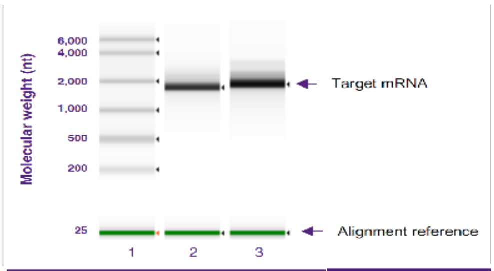

Lane	Sample	Volume
1.	ssRNA ladder (Agilent)	6 µL
2.	2022-31_M1 mRNA IVT (unpurified)	6 µL
3.	2022-31_M1 Final Sample (purified)	6 µL

Volume indicates total volume loaded onto the screentape,
Including 5.0 μL of RNA Loading buffer per 1 μL diluted sample

B.

Sample	Conditions	RIN	Conc. (ng/µL)
SCH_01 (N1-UTP)	4°C 1w	10	179
SCH_01 (N1-UTP)	RT 1w	10	125
SCH_02 (5methoxy-UTP)	4°C 1w	8.2	134
SCH_02 (5methoxy-UTP)	RT 1w	8.3	153
SCH_03 (WT UTP)	4°C 1w	10	169
SCH_03 (WT UTP)	RT 1w	9.8	153
SCH_04 (10% mod. ATP)	4°C 1w	10	188
SCH_04 (10% mod. ATP)	RT 1w	10	170
SCH_05 (25% mod. ATP)	4°C 1w	10	199
SCH_05 (25% mod. ATP)	RT 1w	10	177
SCH_06 (50% mod. ATP)	4°C 1w	10	186
SCH_06 (50% mod. ATP)	RT 1w	9.9	149

C.

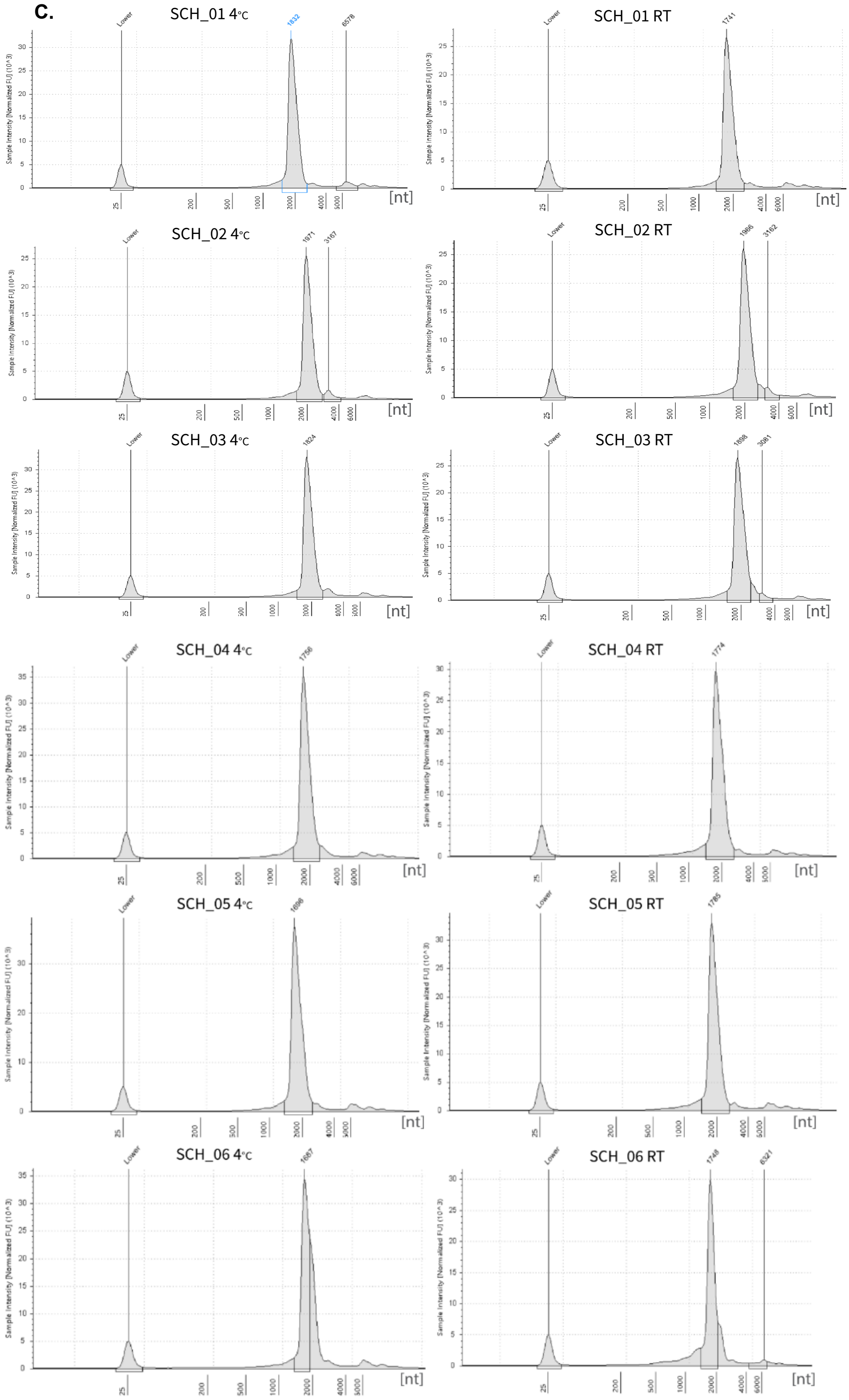

Supplement: Supplementary file 4 — FIGURE S4. Validation of size and stability at 4°C and room temperature of purified mRNA sequence produced by UQ BASE facility using RNA screentape. (A) Electropherogram output from the Agilent ScreenTape assay with corresponding samples in lanes 1–3 showing mRNA integrity for unpurified and purified samples. Distinct bands corresponding to the expected target mRNA sizes are present, indicating successful transcription and purification. Volume in table indicates total volume loaded onto the screentape, including 5.0 µL of RNA loading buffer per 1 µL of diluted sample. (B and C) Stability of the different modified mRNAs was also confirmed after 1 week at 4°C or room temperature (RT). RNA integrity numbers (RIN) and concentrations (conc.) were calculated from the Bioanalyser. SCH_01 = normal uridine; SCH_02 = methoxyuridine; SCH_03 = N1‐methylpseudouridine (m1ψ); SCH_04 = 10% ATP‐5′‐(α‐thio)‐triphosphate (ATPαS); SCH_05 = 25% ATPαS; SCH_06 = 50% ATPαS. [file CTM2-15-e70140-s004.pdf]
